# Diacylglycerol at the inner nuclear membrane fuels nuclear envelope expansion in closed mitosis

**DOI:** 10.1242/jcs.260568

**Published:** 2023-02-02

**Authors:** Sherman Foo, Amaury Cazenave-Gassiot, Markus R. Wenk, Snezhana Oliferenko

**Affiliations:** 1 The Francis Crick Institute, 1 Midland Road, London, NW1 1AT, UK; 2 Randall Centre for Cell and Molecular Biophysics, School of Basic and Medical Biosciences, King’s College London, London, SE1 1UL, UK; 3 Singapore Lipidomics Incubator, Life Sciences Institute and Precision Medicine Translational Research Program, Department of Biochemistry, Yong Loo Lin School of Medicine, National University of Singapore, Singapore

## Abstract

Nuclear envelope (NE) expansion must be controlled to maintain nuclear shape and function. The nuclear membrane expands massively during ‘closed’ mitosis, enabling chromosome segregation within an intact NE. Phosphatidic acid (PA) and diacylglycerol (DG) can both serve as biosynthetic precursors for membrane lipid synthesis. How they are regulated in time and space and what are the implications of changes in their flux for mitotic fidelity is largely unknown. Using genetically encoded PA and DG probes, we show that DG is depleted from the inner nuclear membrane during mitosis in the fission yeast *Schizosaccharomyces pombe*, but PA does not accumulate, indicating that it is rerouted to membrane synthesis. We demonstrate that DG-to-PA conversion catalysed by the diacylglycerol kinase Dgk1 and direct glycerophospholipid synthesis from DG by diacylglycerol cholinephosphotransferase / ethanolaminephosphotransferase Ept1 reinforce NE expansion. We conclude that DG consumption through both *de novo* and the Kennedy pathways fuels a spike in glycerophospholipid biosynthesis, controlling NE expansion, and ultimately, mitotic fidelity.

## Introduction

The double-membrane nuclear envelope (NE) is a hallmark of eukaryotic cells. It consists of the outer nuclear membrane (ONM) continuous with the ER, and the inner nuclear membrane (INM) facing the nucleoplasm. The NE is perforated by the nuclear pore complexes that regulate the transport and exchange of macromolecules between the cytoplasm and the nucleus. Tight control of nuclear shape and size is essential for proper cell function, and aberrant regulation of nuclear morphology has been implicated in aging and disease (Cantwell and Dey, 2021; Webster et al., 2009). The spherical shape of the interphase nucleus minimizes the amount of membrane material required to make the NE. Changes in lipid biosynthesis, nuclear trafficking and/or INM-chromatin attachments may all lead to deviation from this simplest shape (Arnone et al., 2013; Cantwell and Dey, 2021; Webster et al., 2009).

‘Closed’ mitosis of lower eukaryotes, in which the nucleocytoplasmic integrity is maintained throughout chromosome partitioning, represents a fascinating example of highly regulated NE expansion that is not scaled to nuclear volume increase (Zhang and Oliferenko, 2013). The model fission yeast *Schizosaccharomyces pombe* undergoes ‘closed’ mitosis, whereas its relative, *Schizosaccharomyces japonicus* breaks and reforms the NE (Yam et al., 2011). We have previously linked this divergence to differences in nuclear membrane management. As nuclear volume remains constant in ‘closed’ mitosis, the surface area of the mother nucleus must increase to allow for the formation of two daughter nuclei. The nuclear membrane expands dramatically in *S. pombe* but not *S. japonicus*, necessitating NE breakdown in this organism (Yam et al., 2011). Massive expansion of the NE during mitosis in *S. pombe* is driven by the CDK1-dependent inactivation of an evolutionarily conserved regulator of phosphatidic acid (PA) flux, lipin. This does not occur in *S. japonicus*, likely due to differences in trans-regulation of lipin phosphorylation (Makarova et al., 2016). Lipin inactivation upon mitotic entry presumably causes a block in the conversion of PA to diacylglycerol (DG) and a diversion of PA towards production of glycerophospholipids (GPL), thus triggering NE expansion required for ‘closed’ nuclear division (Makarova et al., 2016).

The subcellular distribution of the lipin substrate PA and its product DG at the NE and the possible implications of changes in their distribution for NE expansion and mitotic fidelity remain unknown. Current models of how the regulation of PA flux controls NE growth are largely based on lipidomics analyses of extracts from cells with constitutively (e.g., gene deletion or mutation) or slowly (e.g.¸ overexpression) modified PA regulation network (Han et al., 2008; Santos-Rosa et al., 2005). Yet, in such cases cells settle at metabolic steady states different from the wild-type, limiting the insight into dynamic events occurring in mitosis.

In the budding yeast, which also undergoes ‘closed’ mitosis, lipin deficiency leads to PA accumulation (Han et al., 2008). It was proposed that such an increase in PA might lead to changes in the biophysical properties of the nuclear membrane (Han et al., 2008). This, together with PA-dependent transcriptional upregulation of glycerophospholipid biosynthetic genes (Loewen et al., 2004) could lead to increased nuclear membrane biogenesis. Yet, this regulation circuitry is not conserved in fission yeasts (Rhind et al., 2011; Rutherford et al., 2022); it is also not clear if lipin inactivation leads to increased PA levels in *S. pombe*. To gain an insight into the mechanisms underlying mitotic NE expansion it is imperative to distinguish between different subcellular pools of PA and DG and probe their fates in normal mitosis and upon deregulation of the relevant biosynthetic pathways.

Here we address these questions by using a combination of imaging, genetic and cell physiological approaches in *S. pombe* and the related fission yeast *S. japonicus*.

## Results

### Development of genetically encoded biosensors to probe subcellular distribution of PA and DG in *S. pombe*

**Figure 1:**
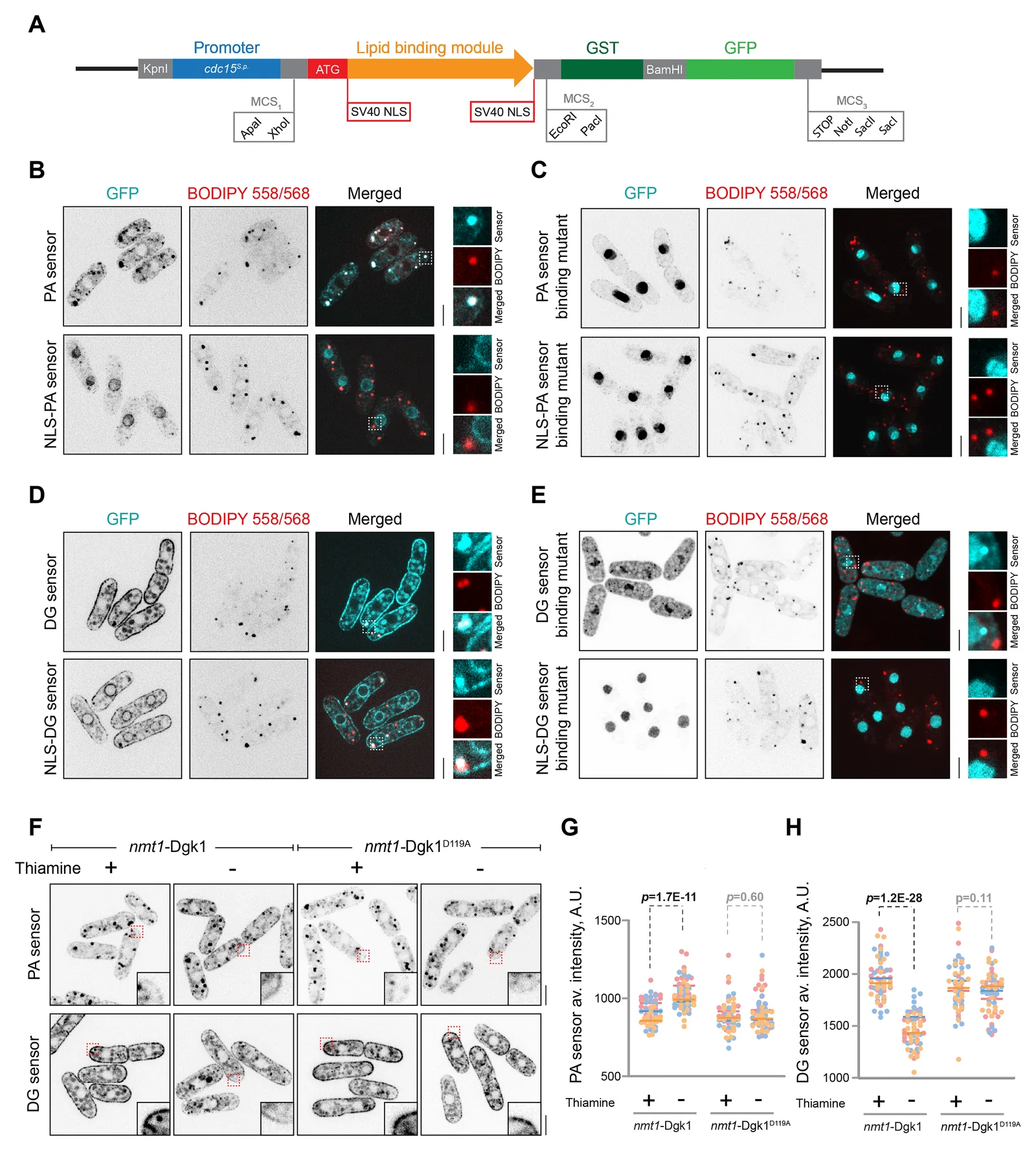
Lipid biosensors for probing subcellular distribution of PA and DG in fission yeast. (**A**) A diagram depicting the design of lipid sensors used in this study. The lipid sensors consist of a lipid-binding module (*S. cerevisiae* Opi1 PA-binding amphipathic helix and *R. norvegicus* PKCβ DG-binding domain) under the control of the *S. pombe cdc15* promoter, with a C-terminal GST-tag and a fluorophore. In the NLS versions of the sensors, the lipid-binding module is flanked by two SV40 NLS motifs. (**B**) Single-plane spinning-disk confocal images of *S. pombe* expressing the PA and NLS-PA sensors show their enrichment at LDs and the INM, respectively. LDs are stained with BODIPY 558/568. (**C**) Mutations in the PA-binding domain abolish normal localization of the PA sensors. Note that a weak bipartite NLS appearing in the mutated PA-binding domain results in its nuclear re-localization in the absence of the SV40 NLS. (**D**) Single-plane spinning-disk confocal images of *S. pombe* cells expressing the DG and NLS-DG sensors show their enrichment at the cortex, LDs and the INM. (**E**) Mutations predicted to abolish DG-binding lead to re-localization of the sensors. The DG-binding mutant sensor becomes largely cytoplasmic whereas the NLS version is enriched in the nucleoplasm. (**F**) Overexpression of the DG kinase Dgk1 increases the intensity of the PA sensor and decreases the intensity of the DG sensor at the cortex. No significant changes are observed upon overexpression of the catalytically inactive D119A mutant of Dgk1. The wild-type and the mutant Dgk1 proteins are expressed under the control of the thiamine-repressible *nmt1* promoter. (**G**) and (**H**) Scatter lots showing the quantitation of these phenotypes; n= 3 biological repeats, 20 cells each. Each biological replicate is color-coded and the medians indicated. p-values are derived using non-parametric heteroscedastic t-test for all data points. In panels (**B**-**F**), insets represent magnified areas indicated by the dotted lines; scale bars represent 5 µm.

We have built a set of genetically encoded PA and DG biosensors, extending the designs used to visualize these lipid classes in *Saccharomyces cerevisiae* (Romanauska and Kohler, 2018). Our PA and DG sensors were expressed from the medium-strength *S. pombe cdc15* promoter and tagged at the C-terminus with a GST biochemical tag and a fluorophore (Fig. 1A). Appending or omitting SV40 NLS sequences around the lipid-binding module has allowed us to probe lipid enrichment in the nucleus or the cytoplasm.

The PA recognition module was engineered from the Q2 domain of the *S. cerevisiae* transcriptional factor Opi1 (Loewen et al., 2004), using further optimization enhancing its PA-binding properties (Hofbauer et al., 2018). Specifically, the PA sensor used throughout this study was constructed by removing the endogenous NLS within the codon-optimized Q2 helix, introducing a G120W point mutation predicted to increase PA-binding selectivity, and duplicating the helix to stabilize it at the membrane (Fig. S1A, B, see Table S1 for the DNA sequence). In exponentially growing *S. pombe*, the cytoplasmic PA sensor was enriched mildly at the cellular cortex and strongly in distinct puncta, confirmed to be lipid droplets (LDs), as visualized by staining cells with the neutral lipid dye BODIPY 558/568 (Fig. 1B, upper panel). The NLS version of the sensor was enriched at the INM (Fig. 1B, lower panel). Confirming sensor specificity, mutation of amino acid residues known to mediate PA binding (Loewen et al., 2004) abrogated the specific localization patterns (Fig. 1C). Note that these mutations yield a weak bipartite NLS and result in nuclear import even in the case of the cytoplasmic construct.

The DG biosensor was developed based on the duplicated codon-optimized *Rattus norvegicus* protein kinase C (PKCβ C1a/b) DG-binding domain (Lucic et al., 2016) (Fig. 1D and Fig. S1C, D, see Table S1 for the DNA sequence). The DG sensor was strongly enriched in the INM and the cellular cortex, as well as in some but not all LDs (Fig. 1D). The DG-binding specificity was validated by using DG-binding deficient versions of the sensor containing two point mutations (Q63E in the C1a domain and Q128E in the C1b domain) (Lucic et al., 2016). The loss of DG binding resulted in the redistribution of constructs to the nucleoplasm and/or cytoplasm depending on the presence of the NLS (Fig. 1E). The cortical localization of the DG biosensor remained unaffected in the absence of ER-plasma membrane attachments in *scs2*Δ *scs22*Δ genetic background (Zhang et al., 2012), indicating that DG is likely enriched at the plasma membrane but not the peripheral ER in *S. pombe* (Fig. S1E).

Of note, significant vacuolar autofluorescence common in fission yeast cells expressing low-to-moderate amounts of GFP-tagged proteins, can be largely corrected by utilizing a recently developed Spectral Autofluorescence Image Correction By Regression (SAIBR) protocol (Rodrigues et al., 2022). Processing images of cells expressing PA and DG sensors by SAIBR highlighted specific subcellular enrichments of these constructs (Fig. S1F).

We validated both sensors further by analysing their distribution under conditions where relative abundance of PA and DG was expected to deviate from the wild-type. Overexpression of the diacylglycerol kinase Dgk1, which catalyses the reverse reaction to lipin by phosphorylating DG to produce PA, from the strong thiamine-repressible *nmt1* promoter resulted in an increase in PA sensor signal at the cellular cortex (Fig. 1F, G), with a concomitant decrease in the cortical intensity of the DG sensor (Fig. 1F, H). These phenotypes were not observed in cells overexpressing the D119A catalytically inactive mutant of Dgk1 (Han et al., 2008).

**Figure 2:**
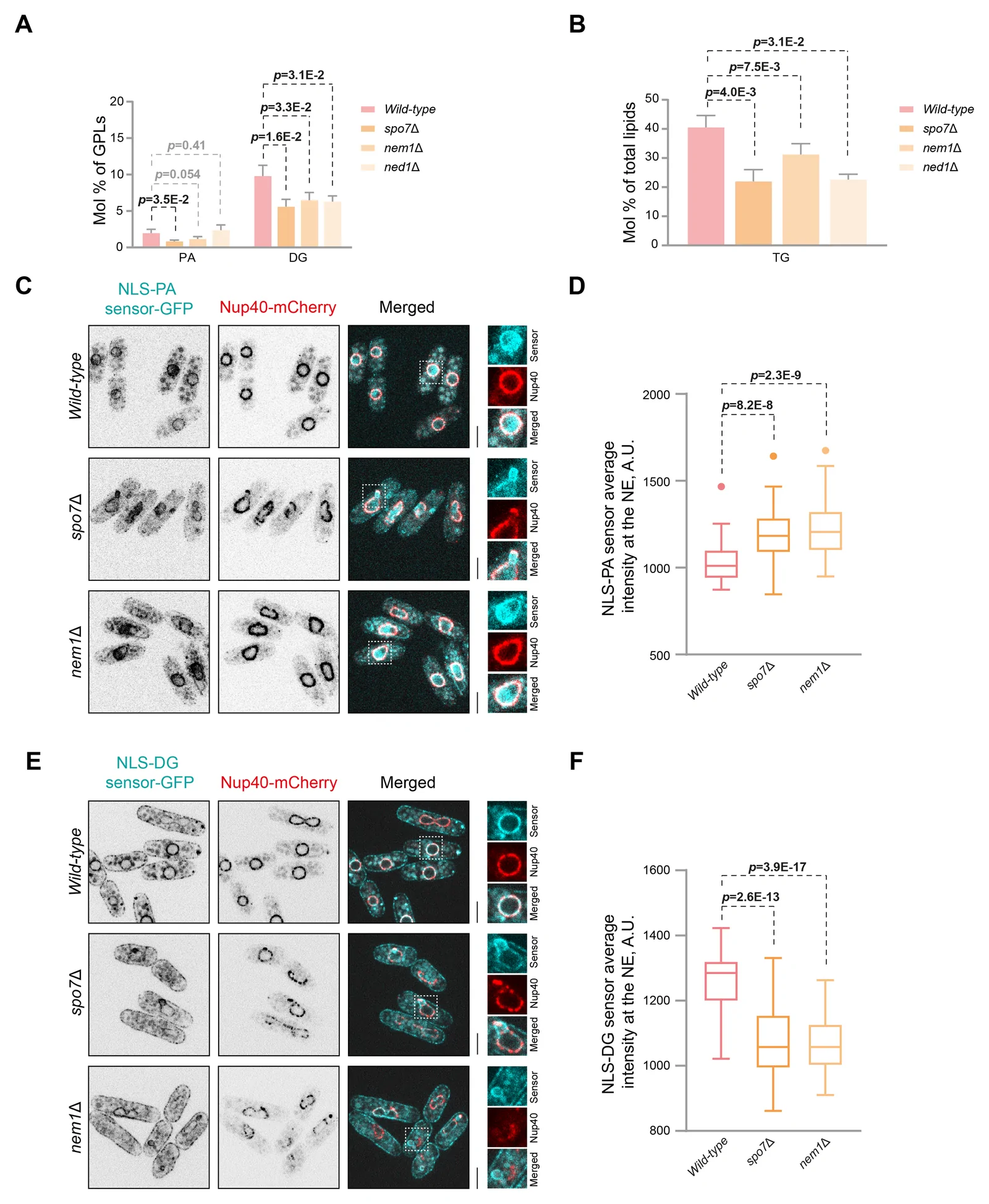
Lipid biosensors are useful to detect changes in PA and DG distribution at the subcellular level. Relative abundance of PA and DG (**A**) and TG (**B**) in *S. pombe* lipin pathway mutants; n=3 biological replicates, results are presented as mean±SD. p-values estimated using non-parametric heteroscedastic t-test. PA sensor levels at the INM are mildly increased (**C, D**) and DG sensor levels are decreased (**E, F**) when lipin is inactive in the absence of its activator phosphatase Spo7-Nem1. Note that whereas the NLS-DG-GFP sensor enrichment at the INM is decreased overall, we detect bright patches at the NE of some nuclei. (**C, E**) Single-plane spinning-disk confocal images of *S. pombe* cells expressing indicated sensors. Nucleoporin Nup40-mCherry marks the nuclear boundary. Insets represent magnified areas indicated by the dotted lines; scale bars represent 5 µm. (**D, F**) n=50 cells, box- and-whiskers plot are created using the Tukey method, p-values are derived from the unpaired t-test.

The phosphatase Spo7-Nem1 positively regulates lipin activity, and the loss of either subunit of the phosphatase complex phenocopies the lack of lipin (Makarova et al., 2016; Santos-Rosa et al., 2005; Siniossoglou et al., 1998). Liquid chromatography electrospray ionization tandem mass spectrometry analyses of total cellular extracts of *S. pombe* lipin pathway mutants revealed a slight decrease in PA levels in the *spo7*Δ mutant and no significant changes in the *nem1*Δ and lipin *ned1*Δ mutants (Fig. 2A). As expected, lipin pathway deficiency resulted in a significant decrease in cellular DG and triacylglycerol (TG) levels (Fig. 2A, B). To investigate these changes at the subcellular level, we analysed the distribution of our sensors in cells lacking the Spo7-Nem1 phosphatase activity. Despite the overall similar or lower cellular abundance, the PA sensor was enriched at the INM of both *spo7*Δ and *nem1*Δ mutant cells grown in the rich YES medium (Fig. 2C, D). The DG sensor levels at the INM were correspondingly lower (Fig. 2E, F).

Of note, the NLS-DG sensor was occasionally enriched at the pronounced NE ‘flares’ formed in *S. pombe spo7*Δ and *nem1*Δ mutants (Fig. 2E, insets). The DG kinase Dgk1 was not excluded from the NLS-DG sensor-rich domains (Fig. S2A, insets). These patches were found throughout the nuclear periphery, i.e., they were not associated with the nucleolus (Fig. S2B) (Witkin et al., 2012). These domains could be also induced by the major reduction in cellular DG through the overexpression of Dgk1 (Fig. S2C), or by increasing the absolute amount of NLS-DG sensor by expressing it under the strong *tdh1* promoter (Fig. S2D). We reason that such local enrichments of NLS-DG sensor may result from the imbalance between the sensor and its target lipid, and care should be taken when designing the experiments aimed at evaluating the suborganellar distribution of DG, particularly in the mutant backgrounds affecting its abundance.

Taken together, our results indicate that the newly developed genetically encoded sensors are useful as reporters to probe changes in PA and DG distribution in live fission yeast cells.

### DG at the inner nuclear membrane is depleted during closed mitosis of *S. pombe*

**Figure 3:**
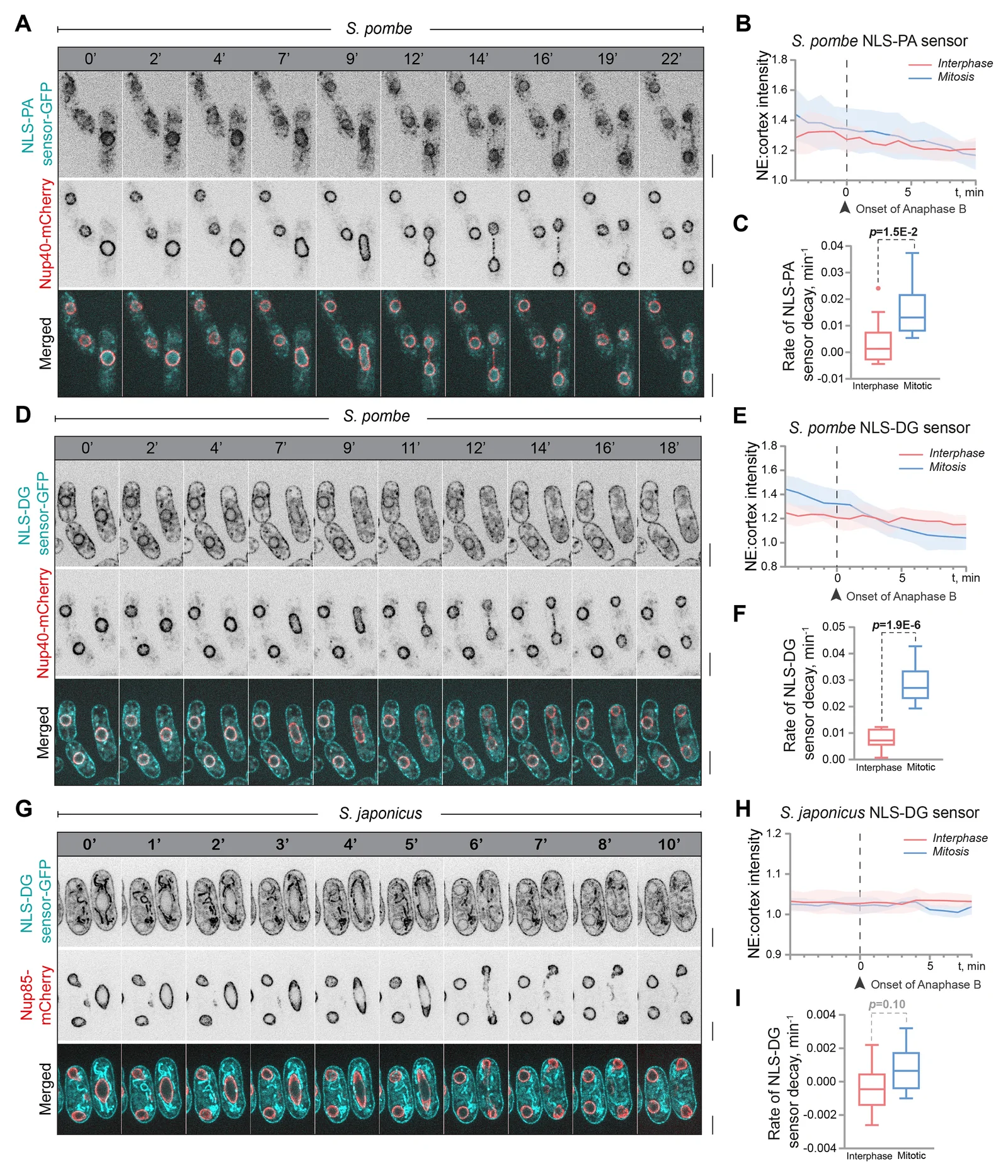
The decay of the DG sensor at the inner nuclear membrane during closed mitosis in *S. pombe* does not result in higher PA sensor enrichment at the NE. (**A**) Time-lapse microscopy montage of mitotic *S. pombe* expressing NLS-PA sensor. (**B**) Average PA sensor intensity at the INM of *S. pombe* are mildly decreasing over the course of mitosis as compared to interphase cells. (**C**) Linear regression shows the rate of the NLS-PA sensor decay in mitotic cells is significantly different as compared to interphase cells. (**D**) Time-lapse microscopy montage of mitotic *S. pombe* expressing the NLS-DG sensor. (**E**) The NLS-DG sensor intensity at the INM rapidly decays as mitosis progresses. (**F**) The rate of decrease of the NLS-DG sensor at the INM of mitotic *S. pombe* is significantly higher than in interphase. (**G**) Time-lapse microscopy montage of the related fission yeast *S. japonicus* that does not expand the NE during mitosis. The NE breakage occurs between 5’ and 6’. (**H**) DG levels at the INM remain relatively constant throughout the semi-open mitosis of *S. japonicus*, quantified in (**I**). (**A, D, G**) Time-lapse spinning disk microscopy; shown are the maximum projections of three Z-slices; scale bars represent 5 µm. Nucleoporins Nup40-mCherry (**A, D**) and Nup85-mCherry (**G**) mark the nuclear boundary. (**B, C, E, F, H, I**) n=10 cells, box-and-whiskers plot are created using the Tukey method, p-values are derived from the unpaired t-test.

Time-lapse imaging showed that the fluorescence intensity of the NLS-PA sensor at the INM decreased mildly during mitosis (Fig. 3A-C). This suggested that lipin inactivation did not cause a spike in PA abundance at the NE as previously proposed for budding yeast (Han et al., 2008). Presumably, upon lipin inactivation all excess PA was diverted for GPL production, necessary for NE expansion (Makarova et al., 2016). The mild decrease could reflect the dilution effect due to the expansion in the surface area of the NE (for review see Zhang and Oliferenko, 2013).

Strikingly, quantitation of the NLS-DG sensor intensity over time revealed that it decreased at approximately twice the rate of the NLS-PA sensor intensity during *S. pombe* mitosis (Fig. 3D-F). To probe if such a decay could be due to changes in sensor abundance, we analysed NLS-DG sensor levels in *S. pombe* cultures undergoing synchronous mitosis by Western blotting. We detected no significant differences in the sensor protein levels over the course of mitosis, indicating that a drop in its fluorescence intensity at the NE was likely reflective of a decrease in DG at that membrane (Fig. S3A-C).

Such a depletion in DG could be due, at least in part, to CDK1-dependent lipin inactivation. In fact, the NLS-DG sensor levels remained constant at the INM of mitotic *S. japonicus* (Fig. 3G-I), as lipin regulation is not tied to the cell cycle in this organism (Makarova et al., 2016). Interestingly, the subcellular enrichment of both PA and DG sensors in *S. japonicus* was different from *S. pombe*. The PA sensor was only very weakly enriched on the NE and the cortex of *S. japonicus* (Fig. S3D), whereas the DG sensor was detected at the cortex and both sides of the NE (Fig. 3G and Fig. S3E). DG levels at the ONM of *S. japonicus* remained stable during mitosis (Fig. S3E-G), similar to its behaviour at the INM (Fig. 3G-I). Similar to *S. pombe*, strong autofluorescence from the vacuoles and mitochondria could be reduced in *S. japonicus* cells expressing the PA and DG sensors by SAIBR correction (Fig. S3H).

Given that *S. japonicus* does not expand the NE during mitosis, our results suggest that the depletion of DG at the INM of *S. pombe* reflects rerouting of the lipid biosynthetic flow towards GPL production necessary for nuclear membrane expansion.

### DG-to-PA conversion by Dgk1 contributes to NE expansion during ‘closed’ mitosis

We next wondered if the DG kinase Dgk1 contributed to the decrease in DG levels at the INM during mitosis of *S. pombe*. Dgk1 was previously identified in *S. cerevisiae* (Han et al., 2008), and was recently shown to display a mild under-expanded ER phenotype (Papagiannidis et al., 2021), hinting at its role in controlling ER growth. The *S. pombe* ortholog is encoded by the *ptp4/dgk1* gene (SPBC3D6.05) (referred to as Dgk1). However, the role of Dgk1 during mitosis and its effect on the subcellular distribution of PA and DG in any system remained unknown.

Consistent with previous observations in *S. cerevisiae* (Han et al., 2008), the loss of Dgk1 in *S. pombe* was able to rescue the NE and ER expansion phenotypes of the lipin pathway mutants (Fig. S4A, B), indicating the overall circuitry conservation. The loss of Dgk1 did not rescue the large lipid droplet phenotype in the lipin-deficient mutants (Fig. S4C) but restored the number of LDs to the wild-type levels (Fig. S4D).

**Figure 4:**
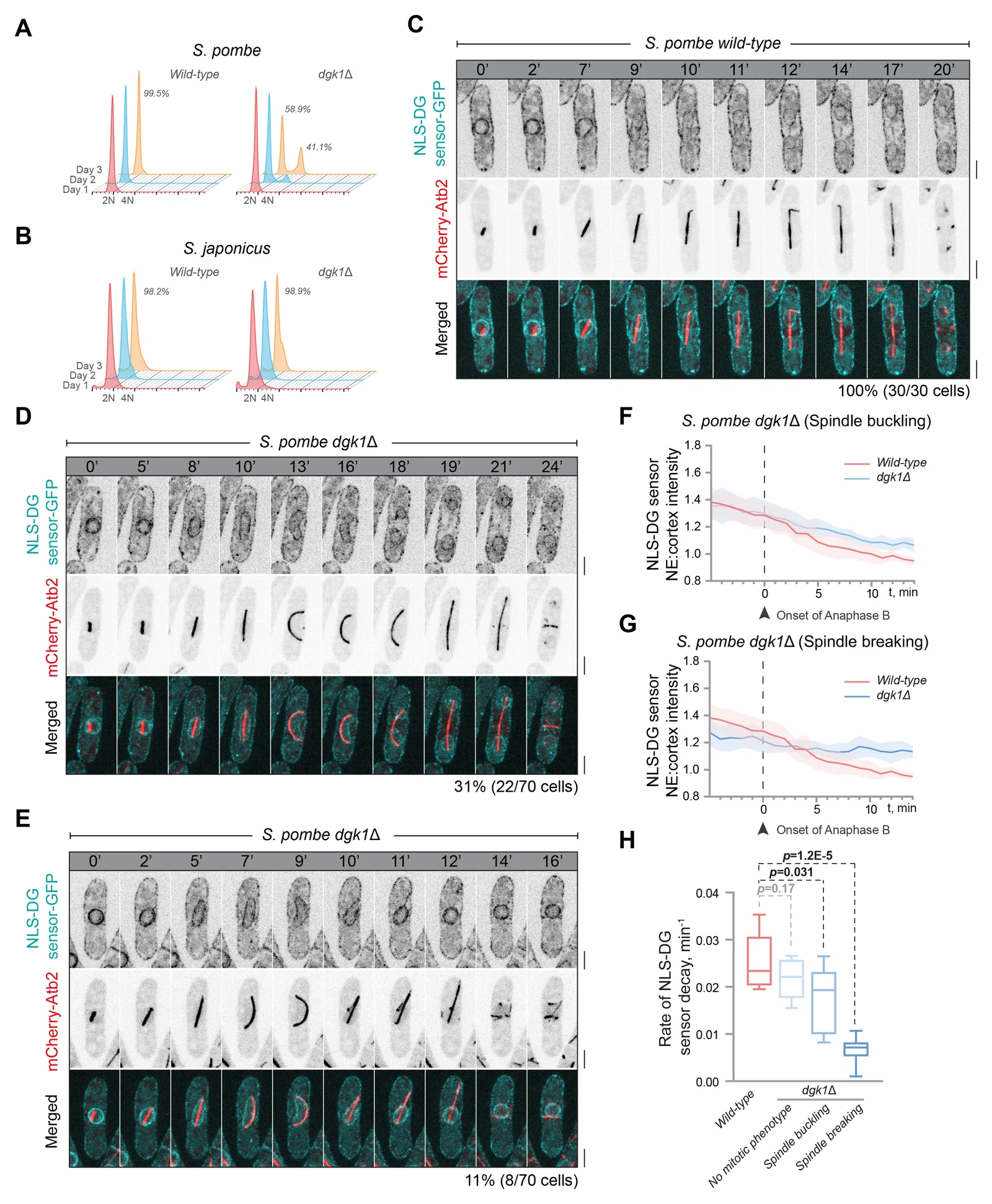
The DG kinase Dgk1 is essential for maintaining mitotic fidelity in *S. pombe*. (**A**) Diploids accumulate upon prolonged culturing of *dgk1*Δ *S. pombe*, as shown by flow cytometric analysis. (**B**) In *S. japonicus*, the loss of Dgk1 does not result in diploidization. (**C**-**E**) Representative time-lapse microscopy montages show a range of anaphase phenotypes in *dgk1*Δ *S. pombe*. (**C**) In the wild-type, the spindle remains relatively straight throughout mitosis, indicating that it is not constrained by the NE. (**D**) 31% of *dgk1*Δ *S. pombe* exhibit anaphase spindle buckling with eventual relaxation, whereas (**E**) 11% break spindles following their compression within the confines of the NE. The decrease in the NLS-DG sensor intensity in cells exhibiting spindle buckling (**F**) and breaking (**G**) phenotypes is compared against the wild-type. (**H**) The rate of decrease of the NLS-DG sensor intensity at the INM is predictive of the severity of mitotic phenotype in *dgk1*Δ *S. pombe*. (**A**-**B**) Shown are representative graphs from at least three biological repeats. (**C**-**E**) Time-lapse spinning disk microscopy; shown are the maximum projections of three Z-slices; scale bars represent 5 µm. α-tubulin mCherry-Atb2 marks the mitotic spindle. The number of cells analysed is indicated at the bottom right of each time-lapse sequence. (**F**-**H**) n=8 cells. The box-and-whiskers plots are created using the Tukey method, p-values are derived from the unpaired t-test.

While analysing *dgk1*Δ *S. pombe* strains we consistently noticed the presence of larger diploids in exponentially growing cultures of haploid cells. Indeed, flow cytometric analysis showed progressive accumulation of diploids in *S. pombe dgk1*Δ cultures (Fig. 4A). In *dgk1*Δ *S. japonicus*, diploidization was not detected (Fig. 4B). We reasoned that if the loss of Dgk1 resulted in a deficiency in NE expansion, the elongating spindle would experience compressive stress along its longitudinal direction, buckle and break, similarly to cells unable to produce fatty acids (Yam et al., 2011) or deficient in mitotic lipin inactivation (Makarova et al., 2016). The spindle collapse would result in the failure to divide the nucleus and diploidization. Time-lapse sequences of mitotic wild-type *S. pombe* co-expressing α-tubulin mCherry-Atb2 and the NLS-DG-GFP sensor, growing in rich medium, showed that the elongating anaphase spindles remained largely straight and DG sensor intensity at the INM decayed (Fig. 4C). Interestingly, the *dgk1*Δ *S. pombe* cells under the same conditions exhibited a range of mitotic phenotypes. Severe buckling of the mitotic spindle was observed in 31% of cells, resulting in bow-shaped nuclear intermediates during anaphase (Fig. 4D). It took longer for these nuclei to divide, and they often formed the daughter nuclei of unequal sizes (Fig. S4E). In 11% of *dgk1*Δ *S. pombe* cells, the NE failed to expand completely, and the mitotic spindle buckled and broke within the confines of the NE (Fig. 4E). Such cells may end up containing the nuclei with twice the genetic content and therefore are the likely source of a diploid cell population in *dgk1*Δ cultures. The remaining 57% of *dgk1*Δ *S. pombe* had no obvious mitotic phenotypes and divided normally (Fig. S4F, G).

Strikingly, we observed a correlation between the rates of DG sensor decay at the INM and the severity of the mitotic phenotypes in *dgk1*Δ *S. pombe* cells (Fig. 4F-H and Fig. S4G). Whereas DG sensor intensity at the INM decayed similarly to the wild-type in the normally dividing *dgk1*Δ mutants (Fig. S4G), it decayed less in cells with buckling spindles (Fig. 4F) and remained constant in those cells that failed to expand the NE (Fig. 4G).

Thus, both the activity of Dgk1 as well as concurrent inactivation of lipin contribute to the decrease in DG levels at the NE during closed mitosis in *S. pombe*. Importantly, this indicates that Dgk1 activity promotes NE expansion required for ‘closed’ mitosis. The fact that DG may decay at different rates in mitotic *dgk1*Δ cells yielding distinct nuclear division phenotypes suggests an underlying metabolic heterogeneity even within isogenic cellular populations.

### The balance between DG and PA but not the elevated levels of PA is the hallmark of NE expansion in *S. pombe*

**Figure 5:**
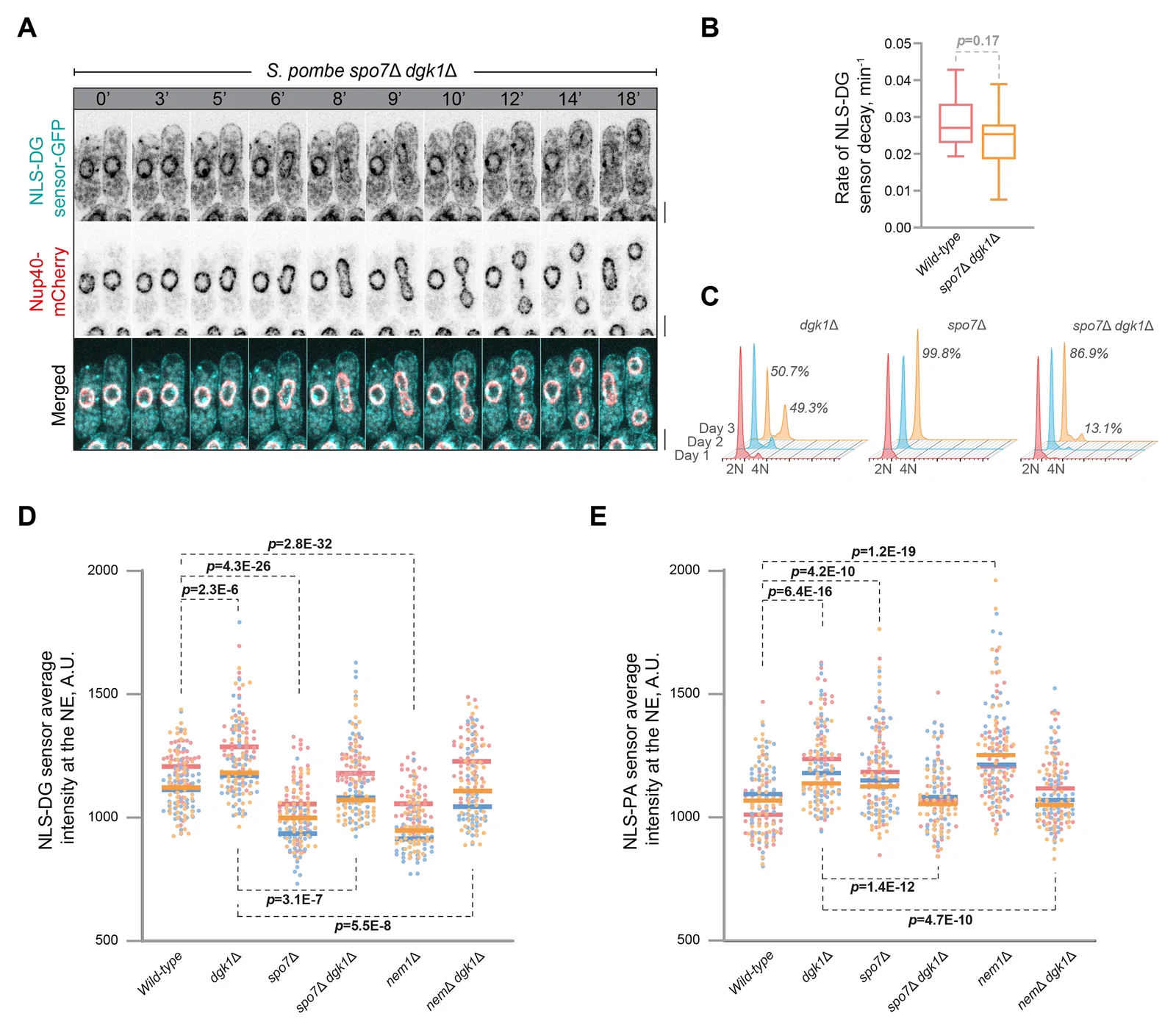
Changes in the PA-to-DG sensor ratio at the INM are consistent with NE expansion in *S. pombe*. **(A)** Time-lapse microscopy of the *spo7*Δ *dgk1*Δ double mutant undergoing mitosis shows a decay of the NLS-DG sensor intensity at the INM, similar to the wild-type. Shown are the maximum projections of three Z-slices. Nucleoporin Nup40-mCherry marks the nuclear boundary; scale bars represent 5 µm. Quantification of the rate of decay is shown in (**B**). n=10 cells; box-and-whiskers plot are created using the Tukey method, p-values are derived from the unpaired t-test. (**C**) Flow cytometry shows that the *spo7*Δ *dgk1*Δ double mutant has a lower proportion of diploids in the population as compared to the *dgk1*Δ cells. Quantification of the levels of DG (**D**) and PA (**E**) sensors at the INM in the indicated genotypes of interphase *S. pombe* are shown as scatter plots; n= 3 biological repeats, 50 cells each. Each biological replicate is color-coded and the medians indicated. p-values are derived using non-parametric heteroscedastic t-test for all data points.

Given that the lack of Dgk1 counteracted excessive NE-ER expansion in the lipin pathway mutants (Fig. S4A, B), we wondered if the loss of lipin activity resulting in a drop in DG and an increase in PA at the NE (Fig. 2D, F), may in turn alleviate insufficient mitotic NE expansion observed in *dgk1*Δ mutant cells. Intriguingly, the *spo7*Δ *dgk1*Δ double mutants displayed normal mitosis and a decay of DG sensor intensity at the NE similar to the wild-type (Fig. 5A, B, see also Fig. S5A, B for *nem1*Δ *dgk1*Δ data). Moreover, we have observed a significant reduction in the diploidization in the cultures of the double mutants compared to the *dgk1*Δ strain (Fig. 5C and Fig. S5C). Inactivating the lipin pathway may constitutively channel PA towards glycerophospholipid synthesis through the CDP-DG pathway, rendering the Dgk1-dependent conversion of DG to PA during mitosis unnecessary.

As expected, Dgk1 deficiency resulted in a mild increase in DG sensor intensity at the NE (Fig. 5D). Perhaps less intuitively, the PA sensor intensity increased substantially in this genetic background, similarly to the lipin pathway mutants (Fig. 5E). This argues that increasing PA levels alone is not sufficient for NE expansion (Han et al., 2008). Instead, we observe changes in the relative abundance of PA and DG sensors at the NE. Indeed, the increase in PA sensor intensity at the INM in the lipin pathway mutants is accompanied by the drop in DG sensor intensity, whereas this is not the case in cells lacking Dgk1. The double mutants lacking both lipin and Dgk1 activities largely restore the relative abundances of PA and DG sensors at the INM (Fig. 5D, E) and show normal NE morphology (Fig. S4A, B). We confirmed that both PA and DG biosensors were expressed at comparable levels in cells of all genotypes (Fig. S5D-G).

Taken together, our results suggest that regulation of DG levels at the INM is important in controlling NE expansion. Yet, how is DG at the NE removed in cells that lack Dgk1 but do not display any mitotic phenotype?

### The Kennedy pathway contributes strongly to glycerophospholipid synthesis required for NE expansion during ‘closed’ mitosis

**Figure 6:**
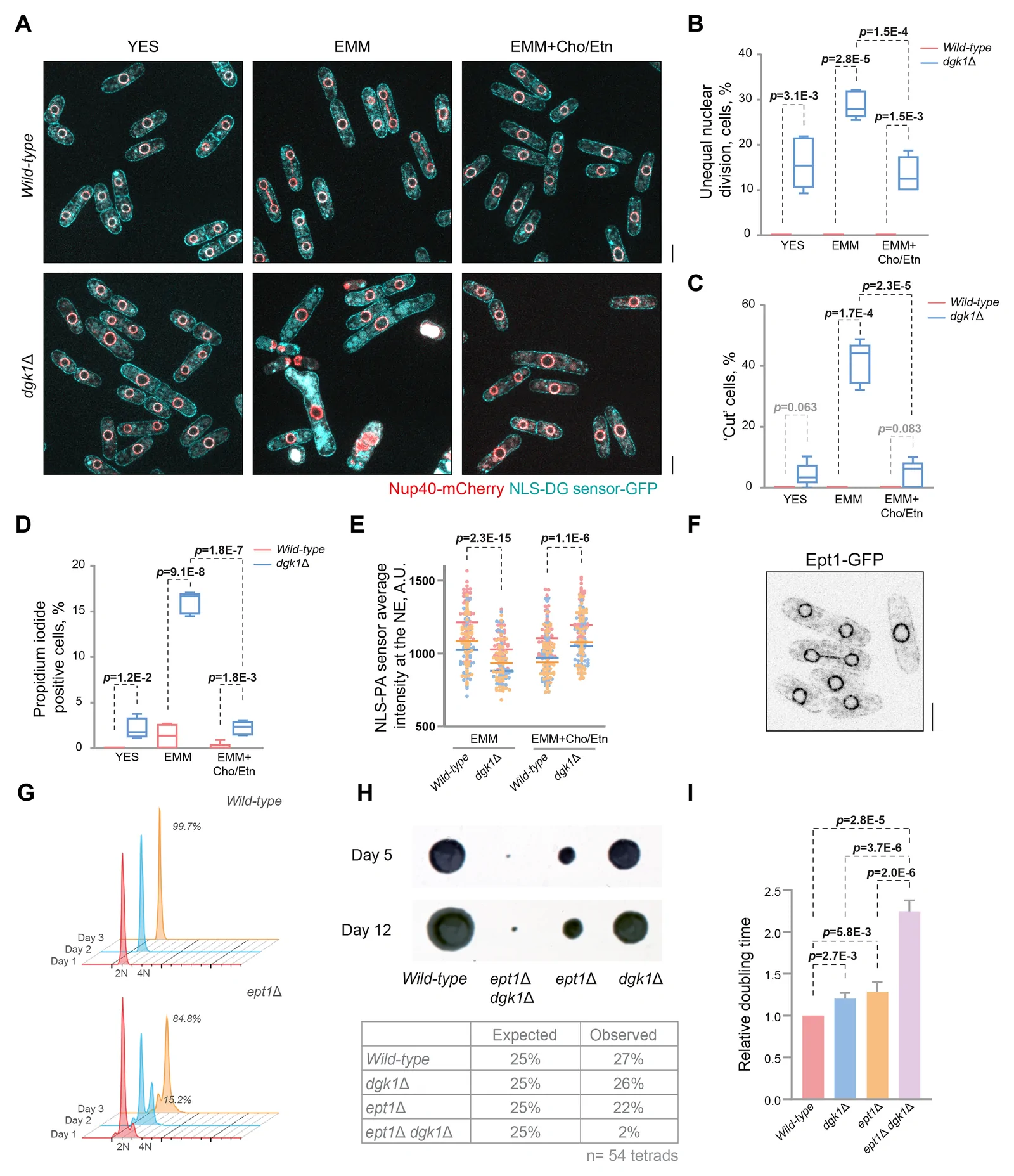
The Kennedy pathway of GPL synthesis collaborates with Dgk1 in sustaining NE expansion required for closed mitosis in *S. pombe*. (**A**) In the absence of the Kennedy pathway precursors, *dgk1*Δ cells exhibit a substantial increase in uneven nuclear divisions (quantitation in **B**) and mitotic failure (‘cut’ phenotype) (quantitation in **C**). Shown are the single-plane spinning-disk confocal images of *S. pombe* cells co-expressing NLS-DG-GFP sensor and Nup40-mCherry. Scale bars represent 5 µm. (**D**) High levels of cell death was detected by propidium iodide staining in *S. pombe dgk1*Δ cells cultured in the absence of the Kennedy pathway precursors. (**E**) Quantification of the levels of PA at the INM in wild-type and *dgk1*Δ cells grown in the indicated medium. Shown are scatter plots; n= 3 biological repeats, 50 cells each. Each biological replicate is color-coded and the medians indicated. p-values are derived using non-parametric heteroscedastic t-test for all data points. (**F**) Single-plane spinning-disk confocal images show Ept1-GFP localization at the NE of *S. pombe*. (**G**) Diploids accumulate upon culturing of the Kennedy pathway deficient *ept1*Δ mutant strain of *S. pombe*, as shown by flow cytometric analysis. (**H**) Tetrad analysis reveals the semi-lethality of the *ept1*Δ *dgk1*Δ double mutant. Shown here is a representative tetrad grown on rich YES agar on day 5 and day 12 after dissection. (**I**) Relative growth rates of the indicated genotypes in rich YES media. (**B, C**) n=4 biological repeats, ≥10 septated cells each; (**D**) n=5 biological repeats, ≥50 cells each; (**H**) n=54 tetrads; (**I**) n=5 biological repeats, error bars represent mean±SD. (**B**-**D**) Box-and-whiskers plot are created using the Tukey method, p-values are derived from the unpaired t-test.

In addition to Dgk1-dependent rerouting of DG towards PA for *de novo* glycerophospholipid synthesis, DG might be also utilized directly by the precursor-dependent Kennedy pathway to produce glycerophospholipids (Carman and Han, 2009b). Our observations suggest that the contribution of the Kennedy pathway is critical for proper nuclear membrane expansion during mitosis. First, *dgk1*Δ mutant cultures grown in the chemically defined medium (EMM) that does not contain choline and ethanolamine (Cho/Etn) precursors required for the Kennedy pathway exhibited a high incidence of cells with unequally dividing nuclei and complete failure in nuclear division (‘cut’ phenotype) (Fig. 6A-C). Many cells in these cultures became non-viable (Fig. 6D). Second, whereas the PA sensor was highly abundant at the INM of *dgk1*Δ cells grown in the presence of the Kennedy pathway (the rich YES medium or EMM+Cho/Etn), we did not observe any enrichment in the absence of the Kennedy pathway precursors (Fig. 6E, compare with Fig. 5E). This suggests that rerouting of DG to GPL biosynthesis via the Kennedy pathway might decrease the consumption of PA via the CDP-DG and/or lipin pathways. Differential activity of the DG-consuming Kennedy pathway within a population of cells could be an underlying cause for the heterogeneity in cellular phenotypes associated with the loss of Dgk1.

Underscoring that the *de novo* and the Kennedy pathways both contribute to glycerophospholipid synthesis, we detected a decrease in NE-ER proliferation in the minimal EMM medium, as compared to either EMM containing Cho/Etn precursors or rich YES medium (Fig. S6A, B). As DG synthesis is decreased in the lipin-deficient mutants, the Kennedy pathway presumably becomes sufficient for consumption of DG at the NE, explaining the mitotic DG decay observed in *spo7*Δ *dgk1*Δ double mutants (Fig. 5A, B; see also Fig. S5A, B for *nem1*Δ *dgk1*Δ data).

Ept1 (SPAC22A12.10) is the diacylglycerol cholinephosphotransferase / ethanolaminephosphotransferase that catalyses the final reaction of the Kennedy pathway in fission yeast. Ept1-GFP is enriched at the NE of *S. pombe* throughout the cell cycle, suggesting that the DG-dependent GPL biosynthesis may occur at this location (Fig. 6F). In order to test directly if the Kennedy pathway contributes to NE expansion, we generated a mutant strain lacking Ept1. The *ept1*Δ mutants exhibited striking diploidization with a majority of the population having double the genetic content within three days of consecutive culture (Fig. 6G). The introduction of the tagged α-tubulin mCherry-Atb2 triggered a much higher incidence of mitotic failure, with mutant cultures swept by diploids even after short period of culturing (Fig. S6C). Importantly, the *ept1*Δ *dgk1*Δ double mutant phenotype was sub-lethal, as observed through genetic crosses and phase-contrast microscopy (Fig. 6H and S6D). Those rare *ept1*Δ *dgk1*Δ double mutants that we were able to recover exhibited severe delay in growth, as compared to the wild-type and the single *ept1*Δ and *dgk1*Δ mutants (Fig. 6I).

In summary, our results show that controlling the utilization of DG at the INM is important for the regulation of NE expansion in *S. pombe*. Both the Dgk1-dependent DG-to-PA conversion and the Kennedy pathway collaborate to channel DG to glycerophospholipid production required for a mitotic spike in NE growth.

## Discussion

**Figure 7:**
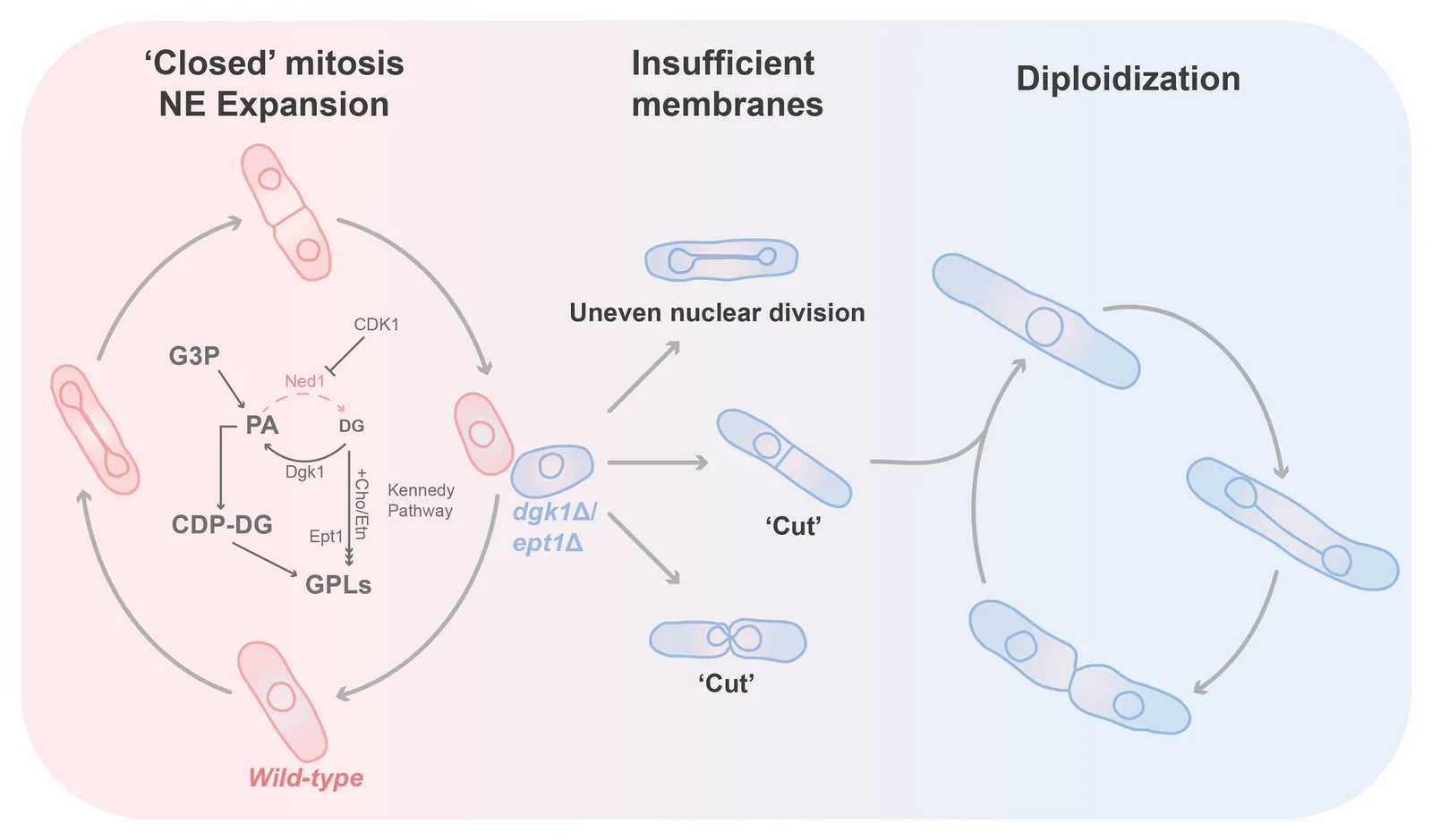
Summary diagram A summary of our hypothesis on how PA and DG flux during NE expansion is controlled in *S. pombe*. In wild-type *S. pombe*, the inactivation of lipin Ned1 by CDK1, together with Dgk1 activity and the Kennedy pathway, channel DG towards GPL biosynthesis. In the absence of Dgk1, insufficient depletion of DG results in reduced membrane availability or/and changes in the biophysical properties of the membrane. When Dgk1 is inactive, cells rely on the Kennedy pathway-dependent DG consumption to produce GPLs. Cell-to-cell heterogeneity in the activity of the Kennedy pathway results in mitotic phenotypes such as uneven nuclear division or chromosome segregation defects (‘cut’ phenotype). Many cells end up with twice the genetic content, forming diploids.

Our results show that NE expansion in *S. pombe* appears to require a pronounced drop in DG levels at the INM. This DG depletion is the result of several enzymatic reactions. Lipin that synthesizes DG from PA is inactivated at mitotic entry by CDK1 (Carman and Han, 2009a; Makarova et al., 2016), reducing DG inflow. At the same time, DG can be either phosphorylated by Dgk1 to produce PA (Han et al., 2008; Kwiatek et al., 2020) or directly used for GPL biosynthesis through the Kennedy pathway (Carman and Han, 2009b). As we do not observe PA sensor levels spiking at the NE at mitosis, it is likely that the Dgk1-produced PA is being used up for the synthesis of membrane lipids through the *de novo* CDP-DG route (Han et al., 2008; Kwiatek et al., 2020) (Fig. 7).

The lipidomics analysis of the *S. cerevisiae* lipin mutant showed an increase in cellular PA levels (Han et al., 2008). Such an increase in PA, a conically shaped lipid capable of generating negative membrane curvature (Zhukovsky et al., 2019), was inferred to modify nuclear membrane properties and lead to the transcriptional upregulation of GPL biosynthetic genes through the Opi1p-Ino2p-Ino4p regulation circuitry (Carman and Henry, 2007; Han et al., 2008), although the latter is not conserved in fission yeasts (Rhind et al., 2011; Rutherford et al., 2022). Of note, PA does not seem to increase neither on global scale in the lipin-deficient *S. pombe* cells (Fig. 2A), nor at the NE during mitosis, when lipin function is inhibited by CDK1 (Fig. 3A, B and (Makarova et al., 2016)). It also does not increase in the budding yeast *spo7*Δ and *nem1*Δ mutants despite the block to lipin activity (Papagiannidis et al., 2021).

Similar to PA, DG has negative spontaneous curvature and was shown to promote fusion of biological membranes, NE assembly, and LD formation (Choudhary et al., 2018; Chung et al., 2018; Domart et al., 2012; Dumas et al., 2010; Miner et al., 2017). It is possible that in addition to fuelling membrane lipid synthesis, depletion of DG or changes in the PA-to-DG ratio may modify the biophysical properties of the NE priming it for expansion.

However, we favour the possibility that rather than having functional implications for NE remodelling, the PA-to-DG ratio at the NE simply reflects the utilization of these lipids by different biosynthetic pathways. Both lipids are precursors for the biosynthesis of other lipid species. GPLs are mainly synthesized through two pathways, the *de novo* CDP-DG-dependent route, which uses PA, and the precursor-dependent Kennedy pathway, which uses DG (Gibellini and Smith, 2010; McMaster and Bell, 1994). In addition, DG can be used for the production of the storage lipid TG (Carman and Han, 2009b; Carman and Henry, 2007; Holic et al., 2020). The differential contribution of these biosynthetic pathways may manifest as changes in the steady state PA-to-DG ratio.

The range of mitotic phenotypes within isogenic populations of Dgk1-deficient cells grown in rich media suggests an underlying metabolic heterogeneity. This may arise due to a number of reasons, e.g., differences in the Kennedy pathway precursor uptake (Gibellini and Smith, 2010), the differential activities of biosynthetic pathways (Stewart-Ornstein et al., 2012), or stochasticity in gene expression (Kaern et al., 2005; Raj and van Oudenaarden, 2008). The phenotypic heterogeneity largely collapses when the Kennedy pathway is disabled either by withdrawal of precursors or the loss of Ept1 (Fig. 6), suggesting that the Kennedy pathway activity may indeed show cell-to-cell variability.

Both Dgk1 and the Kennedy pathway have roles in NE expansion that enables ‘closed’ mitosis and, hence, the maintenance of mitotic fidelity in *S. pombe* (Fig. 4-6; see a diagram in Fig. 7). Interestingly, a functional genomics screen has linked the loss of Dgk1 to meiotic chromosome segregation defects (Blyth et al., 2018). These phenotypes were speculated to be a result of global deregulation in lipid synthesis (Holic et al., 2020). Our data suggest that one possible alternative explanation could be membrane limitation during meiotic remodelling of the NE.

The bulk of the NE membrane expansion during closed mitosis likely occurs *in situ* or at the ER domain close to the NE (Fig. 6F and Fig. S2A). Our results may have implications for mitosis in other organisms. A range of mitotic strategies in nature spans from completely ‘closed’ to completely ‘open’ mitosis, when the NE breaks down in prophase and reforms around the segregated chromosomes upon mitotic exit (Makarova and Oliferenko, 2016). Recent evidence points out at the importance of regulating GPL synthesis for NE reformation and other dynamic events at the NE in metazoans (Bahmanyar and Schlieker, 2020), and it would be of interest to address if the Kennedy pathway or the control of DG-to-PA conversion by diacylglycerol kinases are also critical for these processes.

Advancements in the system-level analyses of lipids have allowed greater scrutiny of their roles in various cellular processes (Han, 2016; Kofeler et al., 2021). Yet, these approaches struggle with informing on lipid dynamics with fine spatiotemporal resolution. Complementing lipidomics and genetics approaches with a set of newly developed lipid sensors for *S. pombe* have allowed us to track changes in the spatiotemporal distribution of PA and DG with subcellular resolution and formulate and test a set of hypotheses on the roles of PA to DG interconversion during mitotic NE remodelling. We believe that these sensors will become an effective and widely used tool for the study of dynamic membrane processes in fission yeasts.

## Materials and methods

### Strains, media, and molecular biology methods

*S. pombe* and *S. japonicus* strains used in this study are listed in Table S2. Standard fission yeast media and methods were used (Aoki et al., 2010; Moreno et al., 1991). All experiments were performed using the rich non-defined YES medium with the exception of lipidomics, thiamine-repressible *nmt1* (Maundrell, 1990) overexpression of Dgk1, and the Kennedy pathway-related experiments shown in Fig. 6A-E and Fig. S6A, B, which were performed using chemically-defined Edinburgh Minimal Medium (EMM). All strains were grown at 30°C unless otherwise specified, in temperature-controlled 200 rpm shaking incubators. Mating was performed on SPA medium and spores dissected on YES agar plates. Homozygous *S. pombe* diploids were generated by *ade6-M210*/*ade6-M216* heteroallelic complementation (Ekwall and Thon, 2017). Choline and ethanolamine (Sigma-Aldrich) were added to EMM at final concentrations of 1 mM.

Molecular genetic manipulations were performed using either a plasmid-based (Keeney and Boeke, 1994) or PCR-based (Bahler et al., 1998) homologous recombination. Lipid sensors were integrated at the *ura4* locus.

Overexpression of Dgk1 and its catalytic mutant was carried out by integration of pREP1-Dgk1 or its mutated version into the *leu1* locus of *S. pombe*. For overexpression experiments, cells were pre-grown in EMM containing 5 µg mL^-1^ thiamine at 30°C for 18 h. These cells were then collected by centrifugation and washed thrice in EMM. Cells were then diluted into separate flasks of EMM with or without thiamine and grown for 20 h prior to imaging.

For experiments in Fig. 6A-E and Fig. S6A, B, cells were precultured in YES at 30°C for 18 h. They were then diluted into separate flasks at OD_595_=0.001 and grown at 30°C for 18 h in the appropriate medium (YES, EMM or EMM+Cho/Etn) prior to imaging the following day. Propidium iodide (Sigma-Aldrich) was used at a final concentration of 0.1 µg mL^-1^.

Growth rates were measured using a VICTOR Nivo Multimode Microplate Reader (PerkinElmer). Cells were initially precultured in YES at 30°C for 18 h. They were then diluted to OD_595_=0.05 and 200 µL of each culture were seeded into 5 wells as technical repeats and grown at 30°C for 50 h without shaking. Absorbance readings were obtained every 10 min. Doubling time was calculated using the formula $T=(\ln\frac{x}{x_{0}})$ where T is the doubling time, x is the final OD_595_ and x_0_ is the initial OD_595_. Results were expressed as doubling time relative to the wild-type.

### Lipid sensor design

All lipid sensors were cloned into the pJK210-based plasmid backbone. DNA sequences of the lipid biosensors were synthesized using GeneWiz FragmentGENE service. *S. pombe* promoters (prom^*tdh1*^, prom^*cdc15*^) were inserted between KpnI-ApaI followed by the biosensor sequence between XhoI-EcoRI. This was immediately followed by the GST tag located between PacI-BamHI in the *S. pombe* sensors, followed by the fluorescent tag (GFP or mCherry) between BamHI-NotI with a stop codon located before the NotI restriction site (Fig. 1A). NLS version of the lipid biosensors contains two SV40 NLS motif appended to both ends of the lipid sensor module.

The PA lipid-binding module consisted of the *S. cerevisiae* Opi1 residues 111-189 to exclude the endogenous NLS signal located at residues 109-112, a duplication of the PA-binding amphipathic helices (residues 114-131) and a point mutation introduced at G120W (Fig. S1A, see Table S1 for the DNA sequence) (Hofbauer et al., 2018).

The DG biosensor consisted of the duplicated DG-binding domain of *R. norvegicus* PKCβ (residues 31-158) (Fig. S1C, see Table S1 for the DNA sequence) (Lucic et al., 2016). PA-binding mutant was constructed with the following mutations: L124R, Y127A, L129R, M131A, I133R, K136A, K137A and R138A (Loewen et al., 2004), while DG-binding mutant contained point mutations at Q63E and Q128E (Lucic et al., 2016). The sequences of all sensor variants were codon-optimized for *S. pombe*.

### Lipidomics

Fission yeast cultures for lipidomics were grown in the defined EMM medium with all supplements (adenine, uracil, histidine and leucine) at 30°C. Cells were collected by filtration and snap-frozen in liquid nitrogen. Lipid extraction was performed using a two-phase chloroform-methanol extraction protocol (Ejsing et al., 2009). Cells were first disrupted in 200 µL of water using a Beadruptor 12 (OMNI international) with ceramic beads at 4.75 speed setting for six cycles of 30 s at 4°C. 100 µL of the diluted lysate was transferred to fresh Eppendorf tubes and 900 µL of chloroform-methanol 17:1 (v/v) was added. The mixture was left shaking at 4°C for 2 h at 200 rpm in an Eppendorf thermomixer shaker. Following that, the mixture was centrifuged at 9000 rpm at 4°C for 2 min. The lower phase was transferred to a fresh Eppendorf tube and dried in a Thermo Fisher Scientific Savant SpeedVac while a second chloroform-methanol 2:1 (v/v) extraction was performed with the upper phase. The second extraction was combined with the first and dried in the SpeedVac before resuspension in 200 µL of chloroform-methanol 1:1 (v/v) containing internal standards (Table S3) and stored at -80°C until further use. Batch quality control samples were made by pooling and mixing 10 µL of each sample lysate. These were aliquoted into 8 Eppendorf tubes for lipid extraction as described earlier. Blanks were prepared by using 100 µL of water in place of lysates for lipid extraction. All lipid extracts from samples were mixed and aliquoted into 9 vials as technical quality control samples. The technical quality control was diluted with chloroform-methanol 1:1 (v/v) to prepare 100, 50, 25, 12.5 and 6.25% diluted samples to assess instrument response linearity.

Initial profiling of *S. pombe* lipids with isotopic correction were performed by Lipid Data Analyzer using the wild-type strain SO2865. Multiple reaction monitoring lists for each lipid class of interest were constructed from the data obtained from the initial profiling experiments and used for targeted LC-MS lipidomics (Tables S4 and S5). All LC-MS/MS was performed on an Agilent 6495A QqQ mass spectrometer connected to a 1290 series chromatographic system with electrospray ionization for lipid ionisation. All sample injection volumes were set at 2 µL. The spray voltage and nozzle voltage were set at 3500 V and 500 V respectively. The drying gas and sheath gas temperatures were maintained at 200°C and 250°C respectively, with flow rates both set at 12 Lmin^-1^. The nebulizer setting was 25 psi. Following instrument stabilisation with 15 injections of a QC sample, instrument stability was monitored by an injection of a QC sample and blank sample every 5 sample injections.

Chromatographic separation of the GPLs phosphatidylinositol, phosphatidylcholine, phosphatidylethanolamine and phosphatidylglycerol was performed using a gradient elution on an ethylene bridged hybrid column with solvent A 1:1 (v/v) acetonitrile/ H_2_O with 10 mM ammonium formate, and solvent B containing 19:1 (v/v) acetonitrile/ H_2_O with 10 mM ammonium formate. The flow rate was set at 0.4 mL min^-1^ with solvent B set at 40% upon injection and increasing to 100% in 7 min. This value was retained for 2 min decreased back to 40% in 1 min, and then retained there until the end of the gradient by 14 min. The eluent was directed to the electron spray ionization source of the mass spectrometer operated in positive ionisation.

Chromatographic separation of PA and phosphatidylserine was performed using a different liquid phase (Triebl et al., 2014). Mobile phase A was deionized water containing 10 mM ammonium formate and 0.5% formic acid. Mobile phase B was 2-propanol/acetonitrile 5:2 (v/v) containing 10 mM ammonium formate and 0.5% formic acid. Gradient elution began at 5% solvent A with a linear increase to 50% over 12 min; the 50% solvent A was held for 3 min, and lastly, the column was re-equilibrated for 15 min.

Chromatographic separation of neutral lipids DG and TG was performed using reversed-phase liquid chromatography on an Agilent rapid resolution HD Zorbax Eclipse-C18 column. The mobile phases A consisted of 3:2 (v/v) H_2_O/ acetonitrile with 10 mmol L^-1^ ammonium formate and B consisted of 1:9 (v/v) acetonitrile/ isopropanol with 10 mmol L^-1^ ammonium formate. The flow rate was set at 0.4 mL min^-1^ with solvent B set at 20% upon injection and increasing to 60% in 2 min, increasing to 100% over 12 min and held there for a further 2 min. This was then decreased to 20% for the next 2 min.

Raw data were processed with MassHunter QqQ Quantitative software (version B.08). Areas under the curve of the chromatogram peaks for each transition were measured and exported to Excel. Normalised peak areas were calculated by dividing the peak areas of the analyte with the corresponding internal standard. Relative abundances were obtained by multiplying the normalised peak areas with the molar concentration of the corresponding internal standard.

All lipidomics experiments were performed with three biological replicates and results presented as mean±SD. Statistical analysis was performed using non-parametric heteroscedastic t-test. All solvents used were LC-MS grade purchased from Sigma-Aldrich or Thermo Fisher Scientific. Annotation of lipid classes and species was done according to the classification system previously described (Liebisch et al., 2013).

### Fluorescence-activated cell sorting

*S. pombe* cells were grown at 30°C in YES medium and 10^7^ cells were collected. Cultures were diluted every 24 h to OD_595_=0.0005, and samples at OD_595_=0.5 were typically collected over the course of three days. Cell pellets were resuspended in 1 mL of 70% cold ethanol and kept at 4°C until required. For staining of cells for FACS analysis, cells were first centrifuged at 6000 rpm for 2 min at 4°C and the supernatant removed. The pellet was washed once in 1 mL of 50 mM sodium citrate and resuspended in 500 µL of 50 mM sodium citrate containing 0.1 mg mL^-1^ RNaseA (Thermo Fisher Scientific). This was incubated at 37°C for 18 h with gentle shaking. 500 µL of 50 mM sodium citrate containing either 2 µM SYTOX green (Thermo Fisher Scientific) for *S. pombe* samples or 8 µg mL^-1^ propidium iodide (Sigma-Aldrich) for *S. japonicus* samples was added, and the suspension sonicated briefly to remove cell doublets before FACS analysis. All FACS experiments were performed on a LSRFortessa Cell Analyzer (BD Biosciences) at the Crick Flow Cytometry Science Technology Platform, with at least three biological replicates.

### Quantification and statistical analyses

Image processing and quantifications were performed in Fiji (Schindelin et al., 2012). Circularity of the nucleus was calculated using the formula $\theta =4\pi \frac{A}{p^{2}}$ where θ is circularity, A is the area and P is the perimeter.

Quantification of PA and DG sensor intensities at the INM or cortex of mitotic cells were obtained using the segmented line function in Fiji with a line width of 5 pixels every 1 min of the time-lapse images. Lipid sensor decay at the NE of mitotic cells was normalized by taking the ratio of the sensor intensity at the NE to the cortex. The lines and shaded area represent the mean±SD respectively. The rate of sensor decay at the NE was calculated by taking the gradient via linear regression of the sensor decay at the NE in individual cells. For quantification of PA and DG at the INM of interphase cells (Fig. 1G, 1H, 2D, 2F, 5D, 5E and 6E), segmented line function in Fiji with a line width of 5 pixels was used. To account for variation in our sensor expression levels, average whole cell fluorescence intensity was also measured using the polygon function in Fiji, and values above 1.5 interquartile range were discarded.

Scatter plots were used to present the data quantifying NLS-PA and NLS-DG levels in cell populations (Fig. 1G-H, 5D-E, 6E). Each biological replicate was color-coded and the medians indicated. p-values were estimated using non-parametric heteroscedastic t-test for all data points. Box-and-whiskers plots were created using the Tukey method in Prism 7 (GraphPad Software). Lipid sensor protein levels (Fig. S3C, S5E and S5G) were compared using parametric ANOVA (n=3 biological repeats).

### Image acquisition

Working concentration of all BODIPY-based dyes (Thermo Fisher Scientific) used was 1 µM. Time-lapse microscopy of cells undergoing mitosis was performed using cells grown on agar pads (Pemberton, 2014). All images were obtained using a Yokogawa CSU-X1 spinning disk confocal system mounted on the Eclipse Ti-E Inverted microscope with Nikon CFI Plan Apo Lambda 100X Oil N.A. = 1.45 oil objective, 600 series SS 488 nm, SS 561 nm lasers and Andor iXon Ultra U3-888-BV monochrome EMCCD camera controlled by Andor IQ3 or Fusion software. Temperature was maintained at 30°C, unless otherwise specified, using an Oko Cage Incubator. Maximum intensity projections of 3 Z-slices of 0.5 µm step size images are shown in time-lapse montages.

For autofluorescence correction, images of PA and DG biosensors in wild-type *S. pombe* and *S. japonicus* (Fig. S1F and S3H) were processed using Spectral Autofluorescence Image Correction By Regression (SAIBR) Fiji plug-in (Rodrigues et al., 2022).

### Cell cycle synchronization and Western Blotting

*S. pombe cdc25-22* temperature sensitive mutant cells expressing NLS-DG sensor were grown for 18 h at the permissive temperature of 24°C in YES medium until OD_595_=0.2-0.3. They were next shifted to the restrictive temperature of 36°C for 3.5 h. Cells were released from the G2/M block by rapid cooling in an ice-water bath to 24°C and transferred to a shaking incubator set at 24°C. 5 OD_595_ equivalents of cells were collected every 20 min and snap-frozen in liquid nitrogen for Western blotting. Further 5 mL aliquots of cells were fixed in 70% ethanol, and cell cycle synchronization was verified by measuring the number of binucleated and septated cells by staining DNA with 2 µM SYTOX green (Fig. S3A-C; n=3 biological repeats). For comparing expression levels of PA and DG sensors in cells of different genetic backgrounds (Fig. S5D-G) 5 OD_595_ equivalents of cells were collected at OD_595_ = 0.4-0.6.

After collection, cells were pelleted for 1 min at 1000 xg. Cell pellets were snap-frozen in liquid nitrogen and stored at -20°C until further use. For lysis of cells, cell pellets were first washed in 1 mL of ice-cold water and then resuspended in 1 mL of water containing 10% TCA for 1 h on ice. Cells were next pelleted for 10 min at 18213 xg at 4°C and the supernatant removed. The cell pellets were then washed once with 1 mL of ice-cold acetone, pelleted again and dried in a speed-vac for 2 min at room temperature. Cell pellets were then resuspended in 300 µL of lysis buffer (50 mM Tris-HCl pH8.0, 1 mM EDTA, 1% SDS) and transferred into 2 mL Lysing Matrix Y tubes (MP Biomedicals). Lysis was performed using a MP Biomedicals FastPrep24 5G homogenizer for 2 × 15 s at 6.5 m s^-1^ speed in a 4°C cold room, with 150 s interval between cycles. Lysates were separated from the beads by puncturing the bottom of the Lysing Matrix Y tubes using a hot needle followed by spinning down at 500 xg for 3 min at 4°C in a 15 mL falcon tube containing a 1 mL pipette tip. Cellular debris were cleared by further centrifugation in a clean Eppendorf tube for 1000 xg for 5 min at 4°C. Lysates were boiled at 95°C for 5 min followed by addition of 100 µL of 4x NuPAGE LDS sample buffer (Invitrogen) containing 10% β-mercaptoethanol before heating for further 10 min at 65°C. 20 µL of lysate was loaded per lane on a NuPAGE 4-12% Bis-Tris gel (Invitrogen) and ran at 160 V for 1 h. Proteins were transferred at 100 V for 1 h using the wet transfer method onto PVDF membranes. Membranes were blocked for 1 h with TBS intercept blocking buffer (LI-COR Biosciences) followed by incubation overnight at 4°C with mouse α-GFP primary antibodies (1:10,000 dilution, Roche, catalogue no. 11814460001). Membranes were washed thrice with 0.05% TBS-Tween followed by incubation with IRDye 800CW IgG conjugated goat anti-mouse secondary antibodies (1:10,000 dilution, LI-COR Biosciences, catalogue no. 926-32210) for 1 h. Proteins were detected using the Odyssey Infrared Imaging System (LI-COR Biosciences). GFP-tagged sensor levels were normalized using mouse anti-β-actin (1:10,000 dilution, Abcam, catalogue no. ab8224) and expressed relative to the wild-type. Quantifications of band intensities were performed on the LI-COR Image Studio software.

## Supplementary Material

Supplementary information

## Summary

Mitotic fidelity in the fission yeast *S. pombe* that undergoes closed mitosis requires diacylglycerol consumption fuelling a spike in glycerophospholipid biosynthesis to enable nuclear membrane expansion.
