# Supplementary material for: Diacylglycerol at the inner nuclear membrane fuels nuclear envelope expansion in closed mitosis
Source: J Cell Sci. Author manuscript; Available in PMC 2026 Aug 26. (PMC7619396; doi:10.1242/jcs.260568)
Supplement: Supplementary file 1 [file EMS217635-supplement-Supplementary_information.pdf]

A

S. cerevisiae (NLS)
Opi1$^{111\text{-}189}$ 2xAH^{G120W}

MAS*(PKKKRKV)*QKL**SRAIAKWKDNL**
Start *SV40 NLS motif* **G120W** **Amphipathic helix motif**
KEYKLNMSRAIAKWKDNLKEYKLNM
G120W Amphipathic helix motif
SIESKKRLVTCLHLLKLANKQLSDKIS

CLQDLVEKEQVHPLHKQDGNARTTT

GAGEDE(PKKKRKV)
SV40 NLS motif

B

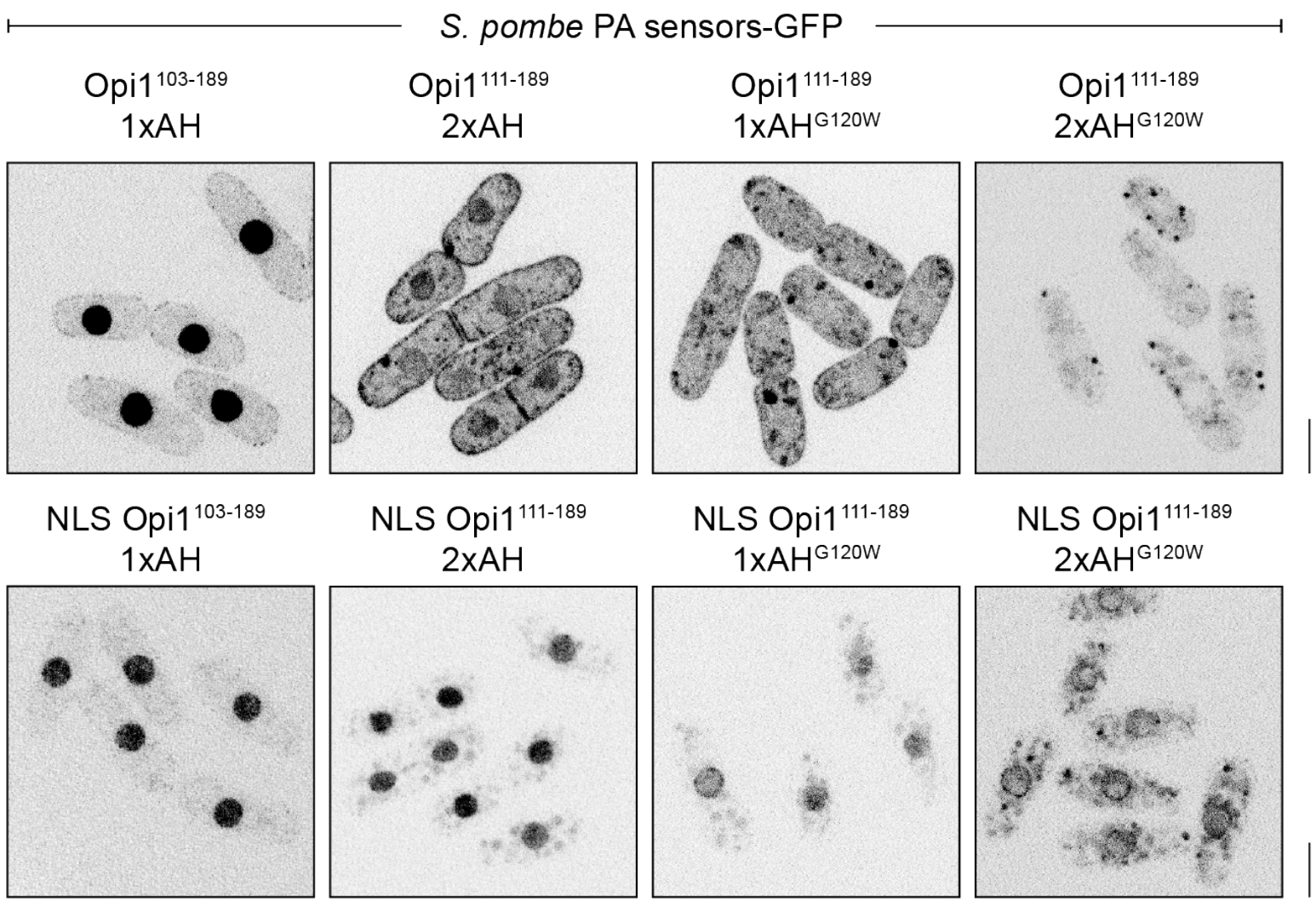

C

R. norvegicus (NLS) 2xPKCβ$^{31\text{-}158}$ DG-Binding Domain

MAS*(PKKKRKV)***VHEVKNHKFTARFFKQPTFCSHCTDFIWGFGKQGFQCQVCCFVVHKRCHEFVTFSCPGADKGPASDDPRSKH**
Start *SV40 NLS motif* **PKCβ DG-binding domain**

KFKIHTYSSPTFCDHCGSLLYGLIHQGMKCDTCMMNVHKRCVMNVPSLCGTDHTERVHEVKNHKFTARFFKQPTFCSHCTDFIW

GFGKQGFQCQVCCFVVHKRCHEFVTFSCPGADKGPASDDPRSKHKFKIHTYSSPTFCDHCGSLLYGLIHQGMKCDTCMMNVHKR
PKCβ DG-binding domain

CVMNVPSLCGTDHTER*(PKKKRKV)*
SV40 NLS motif

D

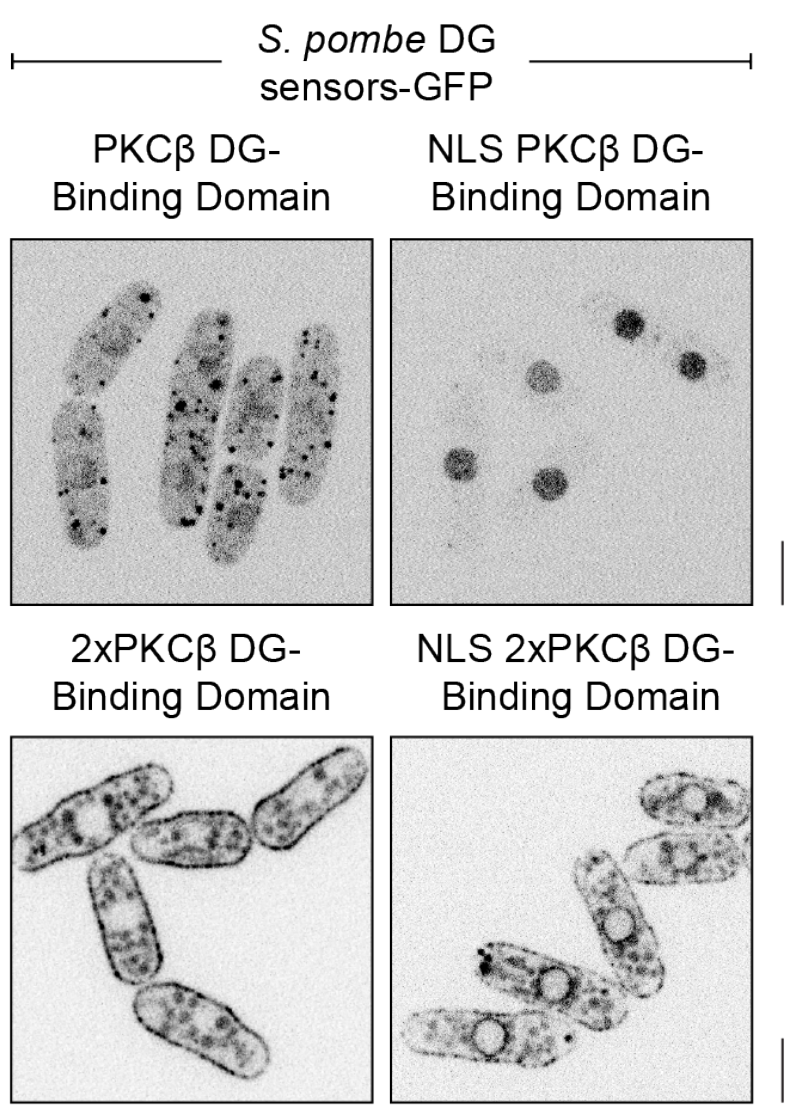

E

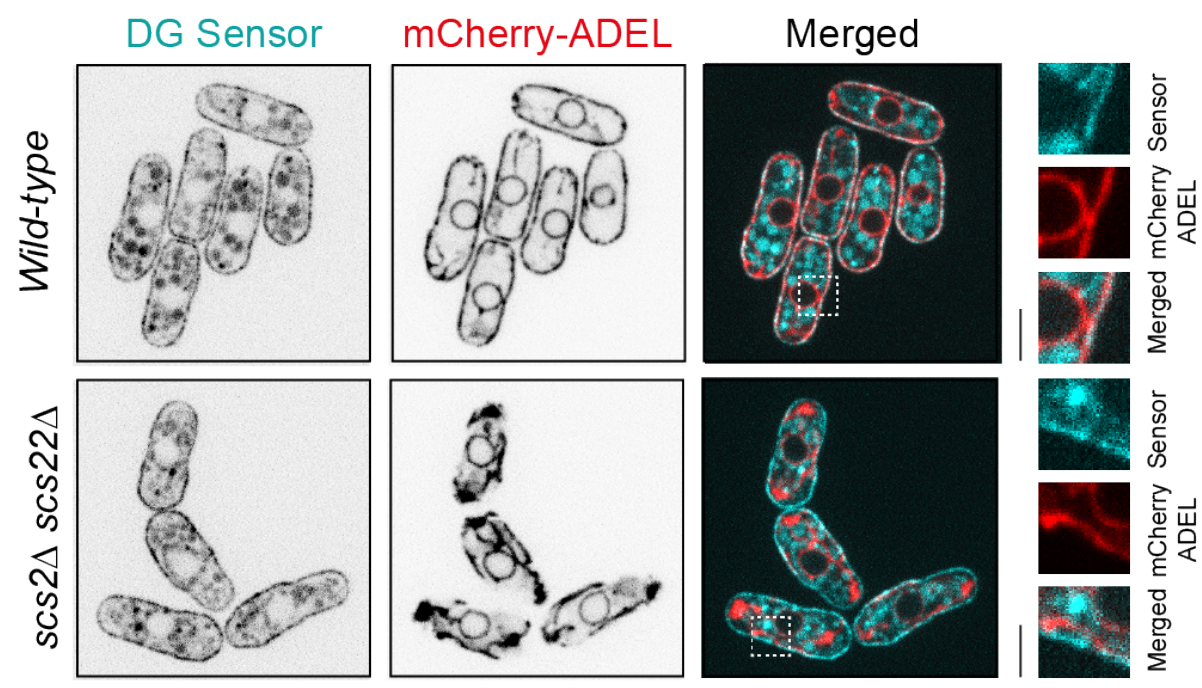

F

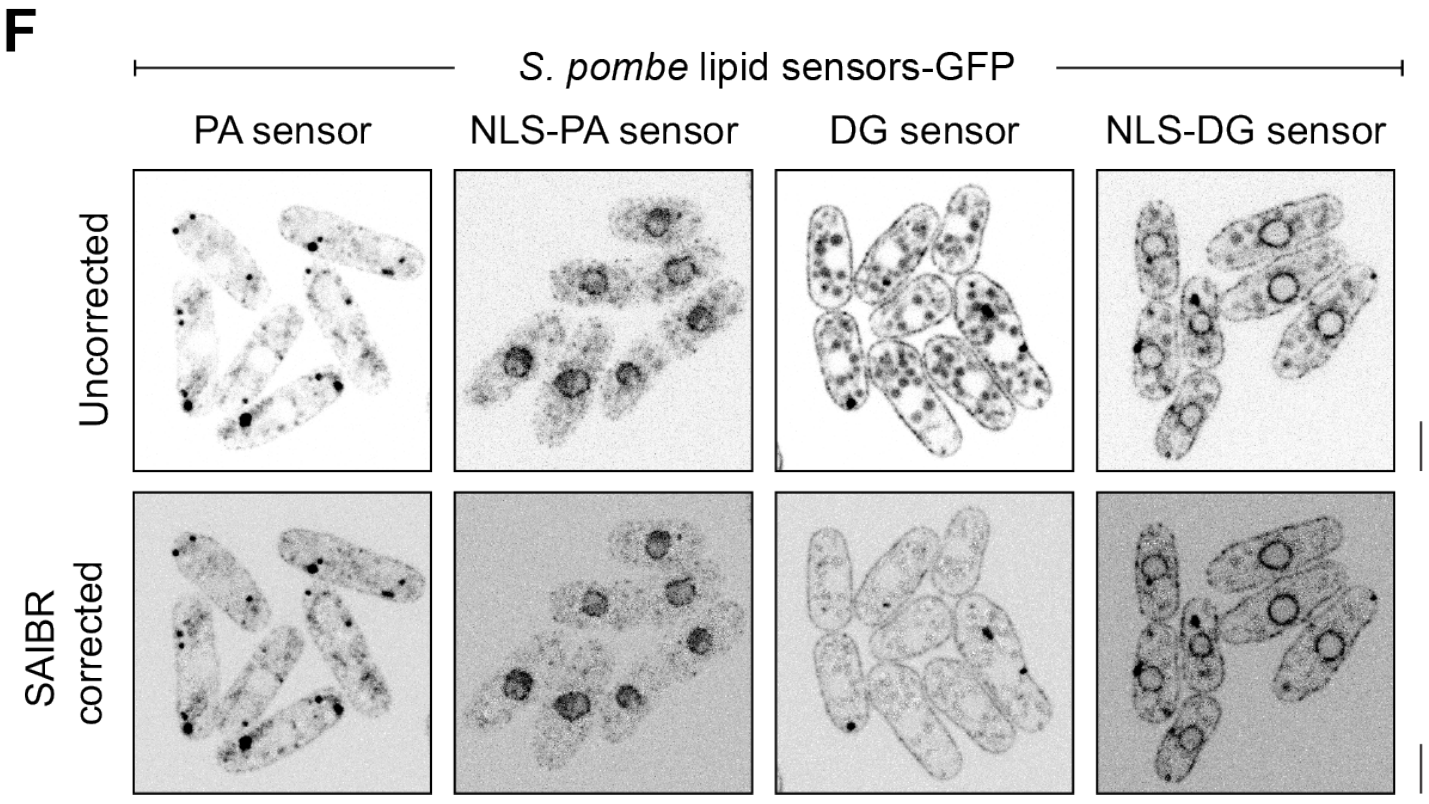

Journal of Cell Science • Supplementary information

Fig. S1. Development and optimization of lipid biosensors in fission yeast. (**A**) Amino acid sequence of the optimized PA lipid sensor module. The optimized PA lipid sensor consists of two consecutive *S. cerevisiae* Opi1 amphipathic helices each containing a G120W point mutation. The amphipathic helices are indicated in bold, mutations indicated in orange, the initiation Met and linker Ala-Ser in red, and SV40 NLS motifs in cyan. (**B**) These single-plane spinning-disk confocal images show optimization of the PA lipid sensors. The single Opi1 amphipathic helix contains an endogenous NLS at residues 109-112. Removal of this endogenous NLS and doubling of the amphipathic helix improves membrane enrichment. Introduction of a single G120W mutation predicted to improve its PA specificity leads to an increased association of the sensors to LDs and the INM. The final iteration of the PA sensors used throughout this work includes two amphipathic helices and the G120W mutation. (**C**) Amino acid sequence of the optimized DG lipid sensor module. The optimized DG lipid sensor consists of two consecutive *R. norvegicus* PKCβ DG-binding domains. The first domain is indicated in bold, the initiation Met and linker Ala-Ser in red, and SV40 NLS motifs in cyan. (**D**) Doubling the PKCβ DG-binding domain leads to membrane enrichment of the DG sensors. (**E**) DG sensor localizes to the cortex in cells where the ER (marked by mCherry-ADEL) is largely detached from the plasma membrane due to the absence of the VAP proteins Scs2 and Scs22. (**F**) Single plane spinning disk confocal images showing *S. pombe* cells expressing indicated sensor constructs before and after autofluorescence correction by SAIBR. (**B**, **D-F**) Scale bars represent 5 μm.

Journal of Cell Science • Supplementary information

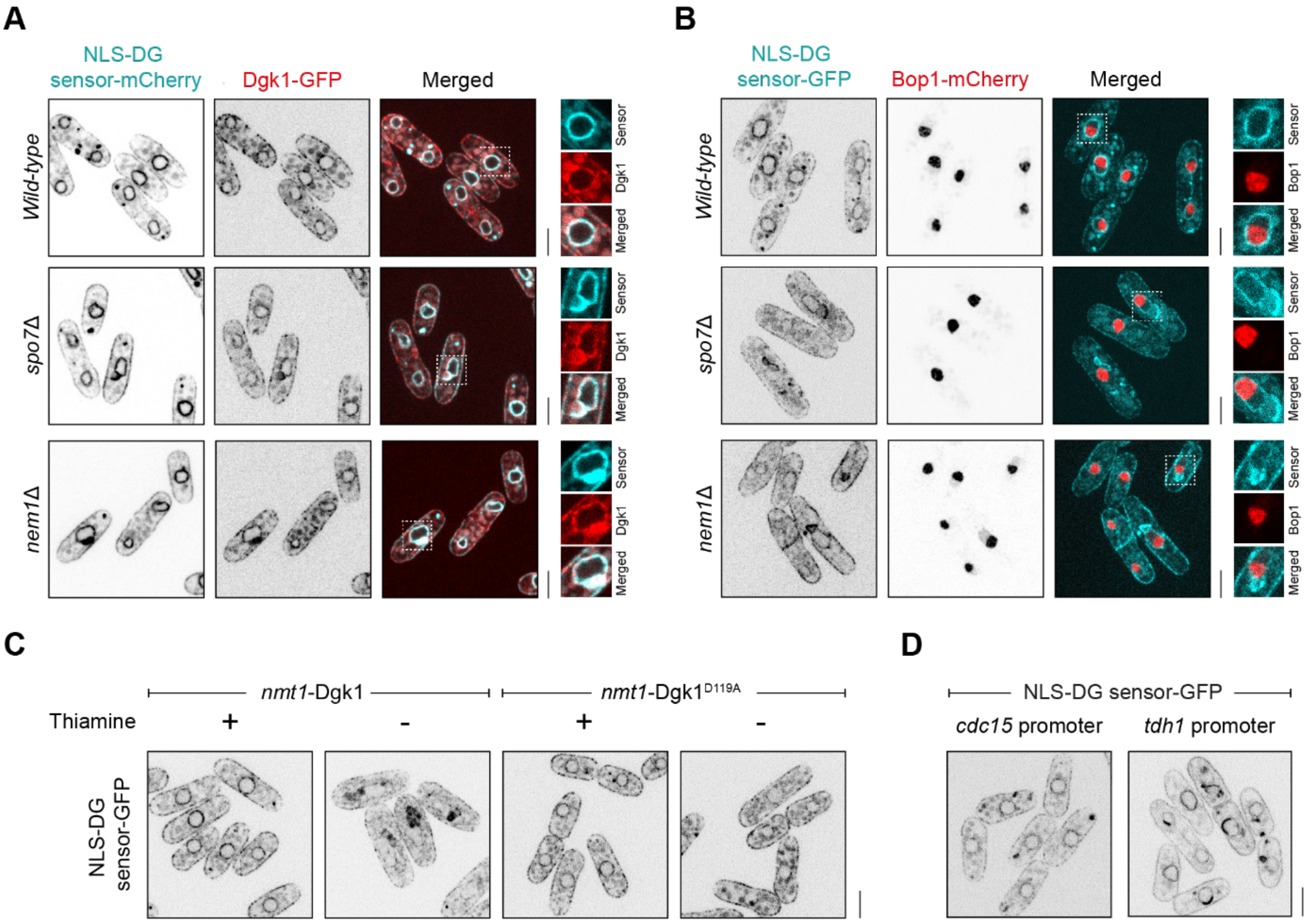

Fig. S2. Excess NLS-DG sensor may lead to its clustering at the INM. (**A**) These single-plane spinning-disk confocal images show that the DG kinase Dgk1-GFP is not excluded from bright NLS-DG-mCherry patches at the INM in *spo7*Δ and *nem1*Δ cells. (**B**) Bright NLS-DG-GFP patches at the INM in YES-grown *spo7*Δ and *nem1*Δ cells are not associated with the nucleolus marked by Bop1-mCherry. These bright patches of NLS-DG-GFP sensor can be also induced by overexpression of Dgk1 causing a drop in DG (**C**) or an increase in the absolute amount of the sensor in a cell by expressing it from a stronger *tdh1* promoter (**D**). The wild-type Dgk1 and the catalytically inactive D119A mutant were expressed under the control of the thiamine-repressible *nmt1* promoter. (**A**, **B**) Insets represent magnified areas indicated by the dotted lines. (**A-D**) Scale bars represent 5 µm.

Journal of Cell Science • Supplementary information

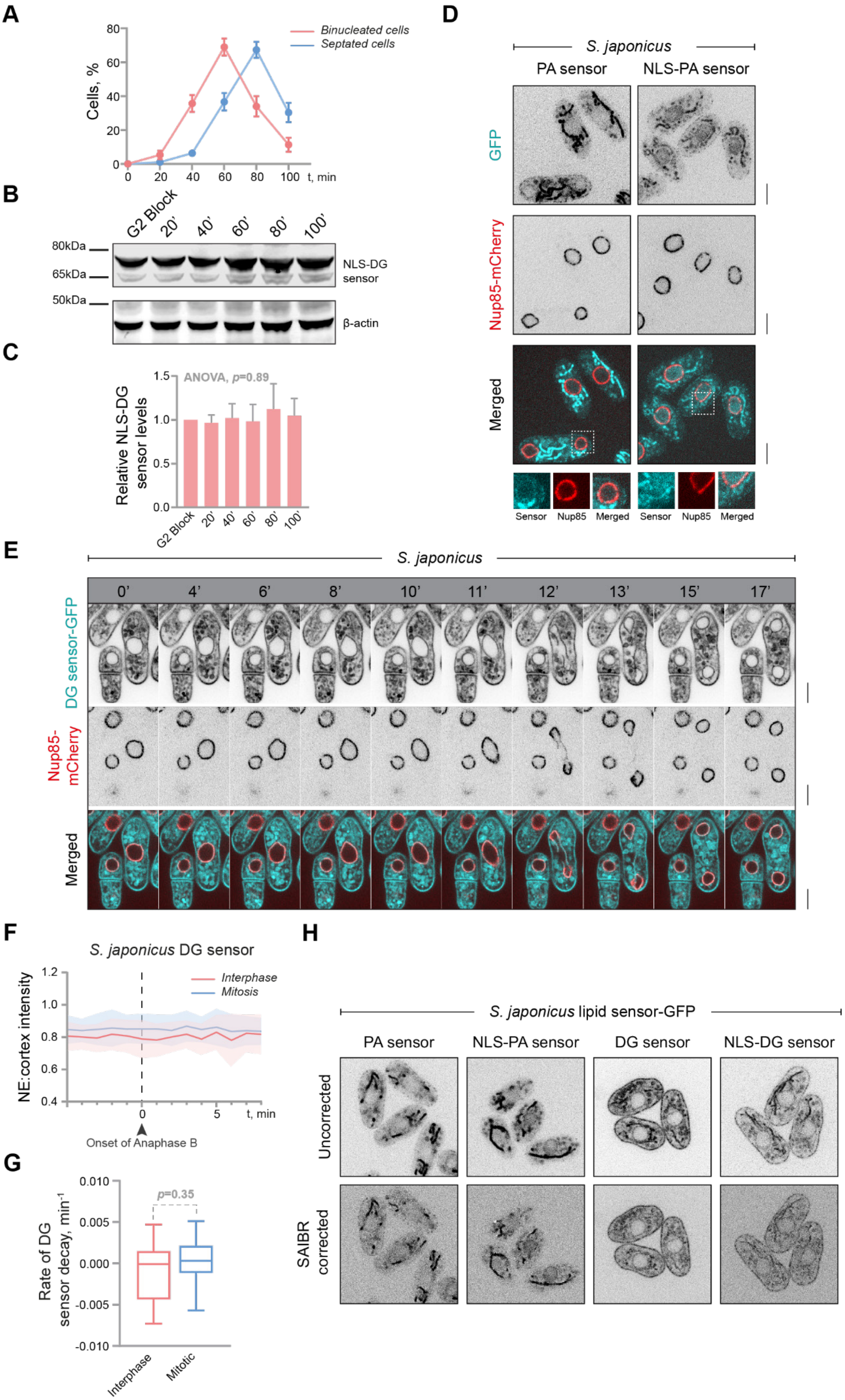

Journal of Cell Science • Supplementary information

Fig. S3. The subcellular distribution of PA and DG in *S. japonicus* differs significantly from *S. pombe*. (**A**) A graph indicating the binucleation and the septation indexes in NLS-DG-GFP sensor expressing *cdc25-22* cultures undergoing synchronous mitoses. n=3 biological repeats, 100 cells each; error bars represent mean±SD. (**B**) Representative Western blots of NLS-DG-GFP sensor and actin of the experiment shown in (**A**). Relevant molecular weight markers are indicated. (**C**) A graph showing relative NLS-DG-GFP protein levels (normalized to actin) in the experiment shown in (**B**). p-value was estimated by parametric ANOVA (n=3 biological repeats). (**D**) Wild-type *S. japonicus*, unlike *S. pombe*, shows a very weak PA sensor enrichment at the INM. (**E**) In addition to the INM, the DG sensor is also enriched at the ONM of *S. japonicus*, as shown in this spinning disk time-lapse microscopy montage. Shown are the maximum projections of three Z-slices. (**F**) The DG sensor intensity at the ONM remains relatively constant throughout the semi-open mitosis of *S. japonicus*, quantified in (**G**). (**H**) Single plane spinning disk confocal images showing *S. japonicus* cells expressing indicated sensor constructs before and after autofluorescence correction by SAIBR. (**D**, **E**) Nucleoporin Nup85-mCherry marks the nuclear boundary. (**F**, **G**) n=10 cells, box-and-whiskers plot are created using the Tukey method, p-values are derived from the unpaired t-test. (**D**, **E, H**) scale bars represent 5 µm.

Journal of Cell Science • Supplementary information

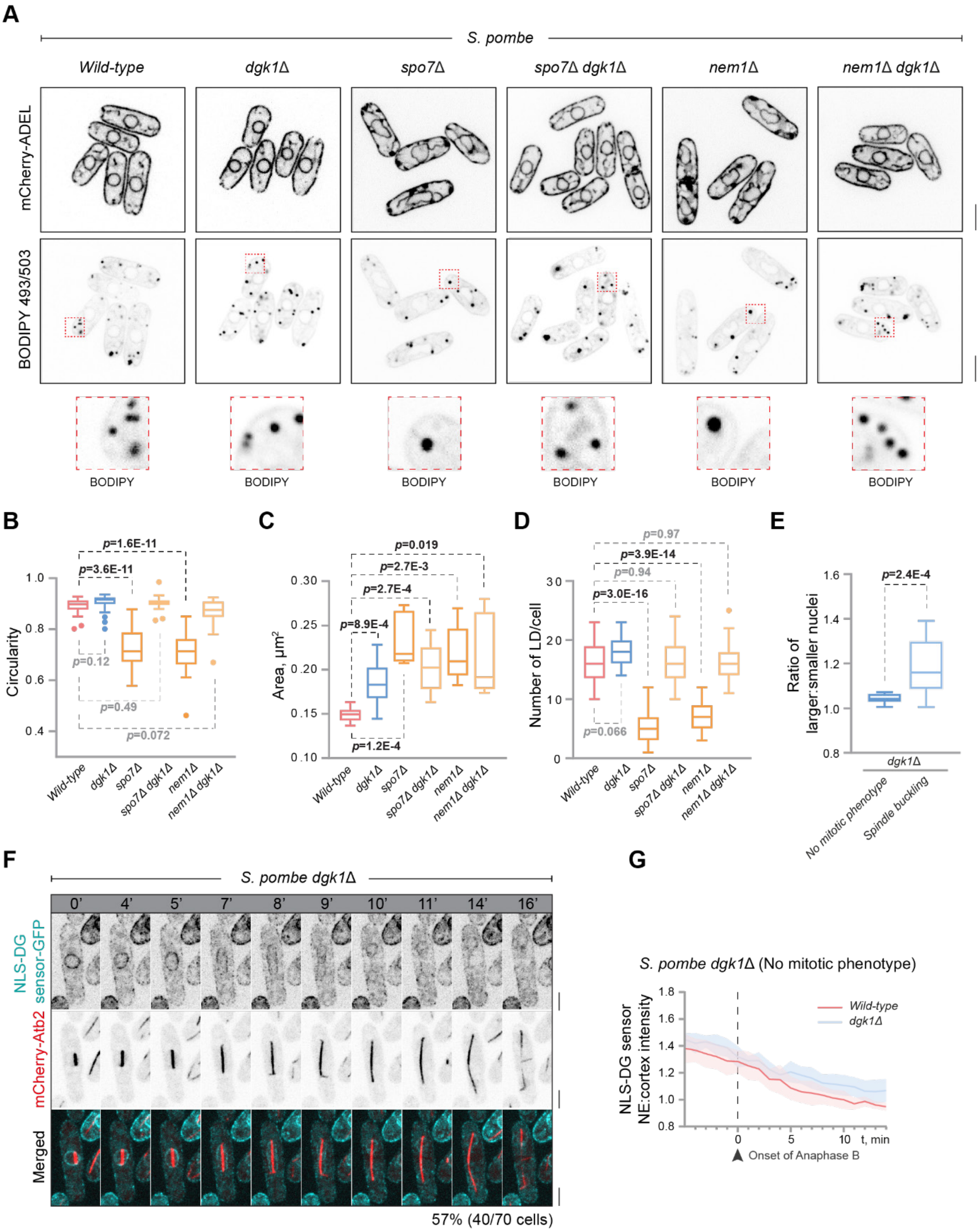

Journal of Cell Science • Supplementary information

Fig. S4. Dgk1 rescues the NE expansion phenotype of the lipin pathway mutants. (**A**) Single-plane spinning-disk confocal images of *S. pombe* of the indicated genotypes expressing the ER-luminal marker mCherry-ADEL and stained with BODIPY 493/503. (**A**-**B**) Loss of Dgk1 rescues the NE proliferation phenotype and the loss of nuclear circularity of *S. pombe spo7*Δ and *nem1*Δ mutants. Interestingly, the loss of Dgk1 does not rescue the large LD phenotype in the lipin mutants (**C**) but restores the average number of LD to the wild-type levels (**D**). (**E**) *dgk1*Δ cells with buckling spindle form unevenly sized daughter nuclei. Shown are the ratios of the larger to smaller daughter nuclei. (**F**) Representative time-lapse microscopy montage of mitotic *dgk1*Δ *S. pombe* that do not display obvious mitotic phenotypes (57% of cells). These cells show a similar decrease in NLS-DG sensor at the INM as compared to the wild-type (**G**). (**A**, **F**) Scale bars represent 5 µm. (**B**-**D**) n=25 cells; (**E**) n=15 cells; (**G**) n=10 cells. Box-and-whiskers plot are created using the Tukey method, p-values are derived from the unpaired t-test.

Journal of Cell Science • Supplementary information

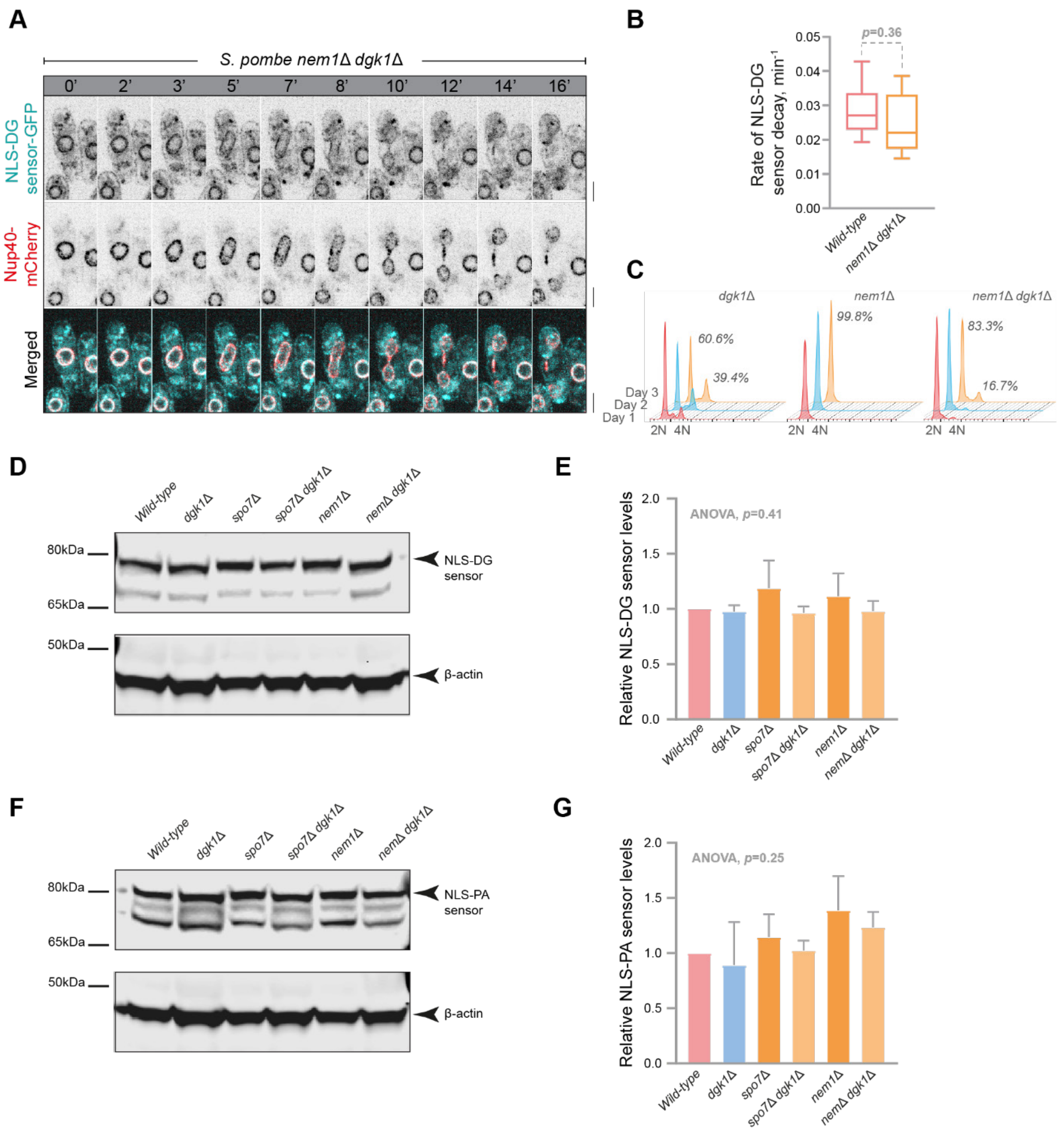

Journal of Cell Science • Supplementary information

Fig. S5. The mitotic phenotype of *S. pombe dgk1*Δ mutants can be rescued by the lack of the catalytic subunit of the Spo7-Nem1 complex, Nem1. (**A**) Time-lapse microscopy showing a similar decrease in NLS-DG sensor intensity at the INM in *nem1*Δ *dgk1*Δ double mutant as compared to the wild-type *S. pombe* during mitosis. Shown are the maximum projections of three Z-slices. Nucleoporin Nup40-mCherry marks the nuclear boundary; scale bars represent 5 µm. (**B**) No significant difference in the rate of NLS-DG sensor decay is observed between the wild-type strain and *nem1*Δ *dgk1*Δ double mutant. Box-and-whiskers plot are created using the Tukey method. (**C**) Deletion of *nem1* rescues the diploidization in *dgk1*Δ *S. pombe* as shown by flow cytometric analysis. Shown are the representative graphs from three biological repeats. Representative Western blots and their quantifications of NLS-DG-GFP (**D**, **E**) and NLS-PA-GFP (**F**, **G**). Relevant molecular weight markers are indicated. Graphs in (**E**, **G**) show relative sensor protein levels normalized to actin. p-values were estimated by parametric ANOVA (n=3 biological repeats).

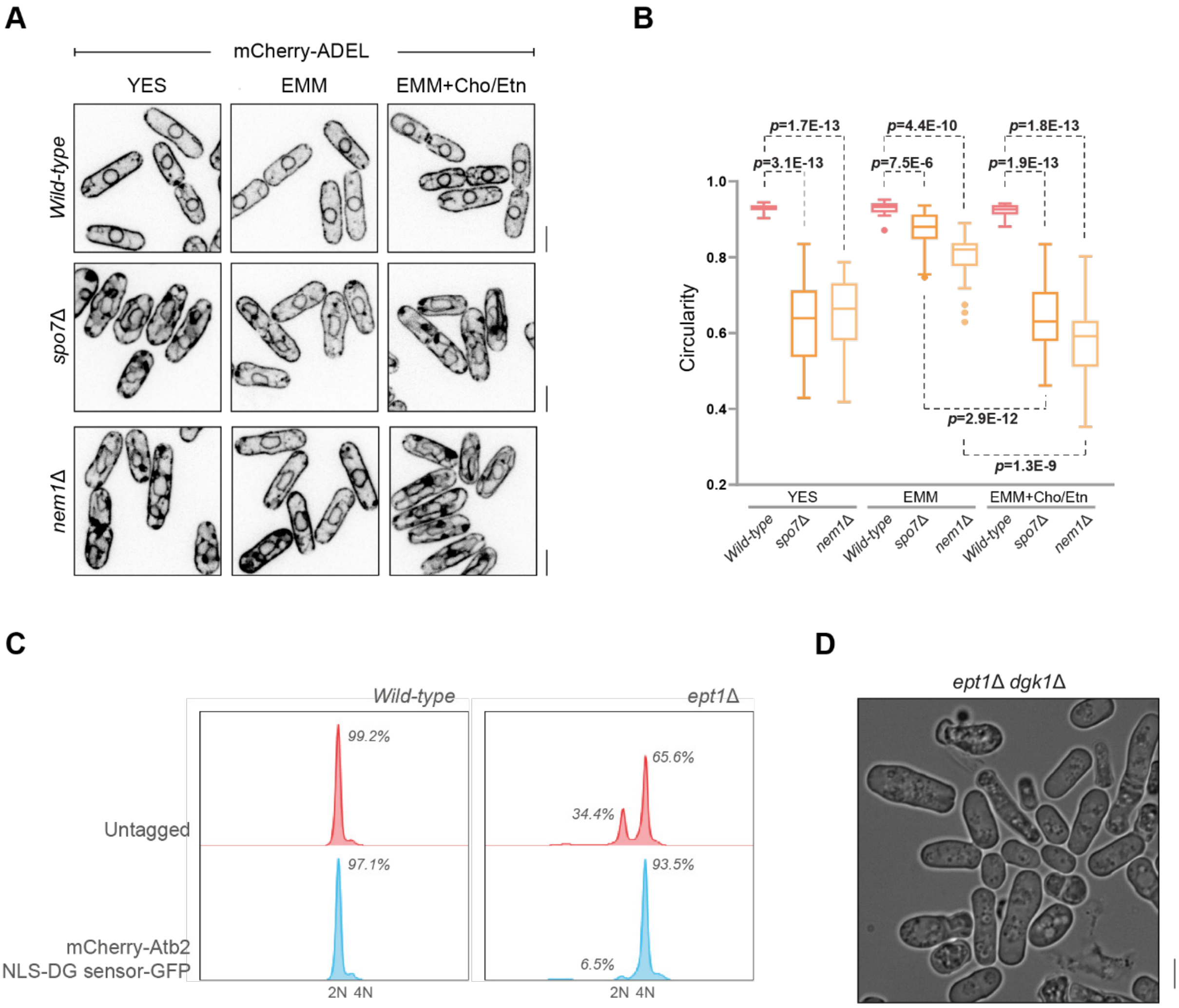

Fig. S6. The Kennedy pathway strongly contributes to GPL biosynthesis in fission yeast. (**A**) Wild-type and lipin mutants expressing the ER-luminal marker mCherry-ADEL shows significantly less NE and ER expansion when cultured in the minimal EMM media. Supplementation of the Kennedy pathway precursors results in increased NE and ER expansion. Scale bars represent 5 µm. Quantifications of nuclear circularity are shown in (**B**). n=25 cells, box-and-whiskers plot are created using the Tukey method. p-values are derived from the unpaired t-test. (**C**) Flow cytometric analysis shows that *S. pombe ept1Δ* mutants expressing α-tubulin mCherry-Atb2 are more susceptible to diploidization compared to the untagged strain, as shown for overnight cultures in rich YES medium. (**D**) High levels of cell death and large cells indicating the presence of diploids can be observed under phase contrast microscopy in the *ept1Δ dgk1Δ* double mutant strain.

Journal of Cell Science • Supplementary information

Table S1. DNA sequences of the optimized PA and DG lipid sensor modules

Click here to download Table S1

Table S2. *S. pombe* and *S. japonicus* strains used in this study

Click here to download Table S2

Table S3. Lipid standards for lipidomics

Click here to download Table S3

Table S4. GPL transition list (targeted lipidomics)

Click here to download Table S4

Table S5. DG and TG transition list (targeted lipidomics)

Click here to download Table S5

Journal of Cell Science • Supplementary information
